# Divergence at the *IRX* gene cluster underlies extreme trophic polymorphism in a cichlid fish (*Herichthys minckleyi*)

**DOI:** 10.1038/s42003-026-09689-6

**Published:** 2026-02-21

**Authors:** C. Darrin Hulsey, Paolo Franchini, Paul Masonick, Andreas Kautt, Gonzalo Machado-Schiaffino, Martin Pippel, Francisco J. García-de León, Eugene Myers, Axel Meyer

**Affiliations:** 1 School of Biology and Environmental Science, University College Dublin, Dublin, Ireland; 2 Department of Biology, University of Konstanz, Konstanz, Germany; 3 Department of Ecology and Biology (DEB), Tuscia University, Viterbo, Italy; 4 Department of Biology, Washington University, St. Louis, MO USA; 5 Department of Functional Biology, Area of Genetics, University of Oviedo, Oviedo, Spain; 6 Systems Biology Center, Max Planck Institute of Molecular Cell Biology and Genetics, Dresden, Germany; 7 Laboratorio de Genética para la Conservación, Centro de Investigaciones Biológicas del Noroeste, La Paz, BCS Mexico; 8 Museum of Comparative Zoology, Harvard University, Cambridge, MA USA

**Keywords:** Adaptive radiation, Evolutionary genetics

## Abstract

The origin of the extensive phenotypic divergence characterizing adaptive radiation could often be geographically localized and genetically simple. In a classic case of a trophically polymorphic cichlid fish (*Herichthys minckleyi*), we investigated alternative genomic processes that could have produced its extreme within-population variation in pharyngeal jaw tooth size. First, we generated a high-quality reference genome for its close relative (*H. cyanoguttatus*) to dissect the genetic architecture of this dental polymorphism. Then, using whole genome resequencing across the small Cuatro Ciénegas valley where *H. minckleyi* is endemic, we found substantial micro-geographic subdivision and effectively no genetic structure due to pharyngeal morphotype. We also employed quantitative trait loci mapping and genome wide association to pinpoint a single peak in an Iroquois-related (*IRX*) gene cluster associated with *H. minckleyi*’s dental divergence. Interspecific introgression in this genomic region appears negligible, suggesting the genomic basis of the polymorphism likely arose within cichlids confined to Cuatro Ciénegas. Because *H. minckleyi* tooth size disparity is comparable to that found in all Central American cichlids, this offers a striking example of how genomic divergence at a single locus could produce a punctuated burst of eco-morphological divergence that generates phenotypic breadth comparable to a highly diverse cichlid adaptive radiation.

## Abstract (web summary)

Genomic dissection combining a novel reference genome, QTL-mapping, and genome-wide association identified a single peak in an Iroquoisrelated (IRX) gene cluster as underlying the extreme dental polymorphism in the cichlid fish *Herichthys minckleyi*.

## Introduction

Dissection of the genomic basis underlying trophic polymorphism can provide a mechanistic view into how population-level ecological divergence fuels adaptive radiation^1–3^. For example, cichlid fishes exhibiting polymorphic jaws could offer a genomic snapshot of what factors contribute to the early stages of ecological speciation^4^. However, these types of polytypic species might characterize lineages that are in the process of collapsing into a single panmictic population^5–7^. Alternatively, hybridization could be constructively aiding the initial stages of adaptive radiation and could even be the source of novel polymorphic phenotypes^8–14^. Then again, the genomic changes that are able to generate and maintain the astounding phenotypic divergence present in some polymorphic species could be relatively simple^3,4^. To delineate among several alternatives that could shape the trophic variability in an iconic cichlid fish, we used a newly generated cichlid reference genome, quantitative genetics, and whole-genome population sequencing to determine the mechanisms generating an extreme trophic polymorphism in the cichlid fish *Herichthys minckleyi*.

**Fig. 1:**
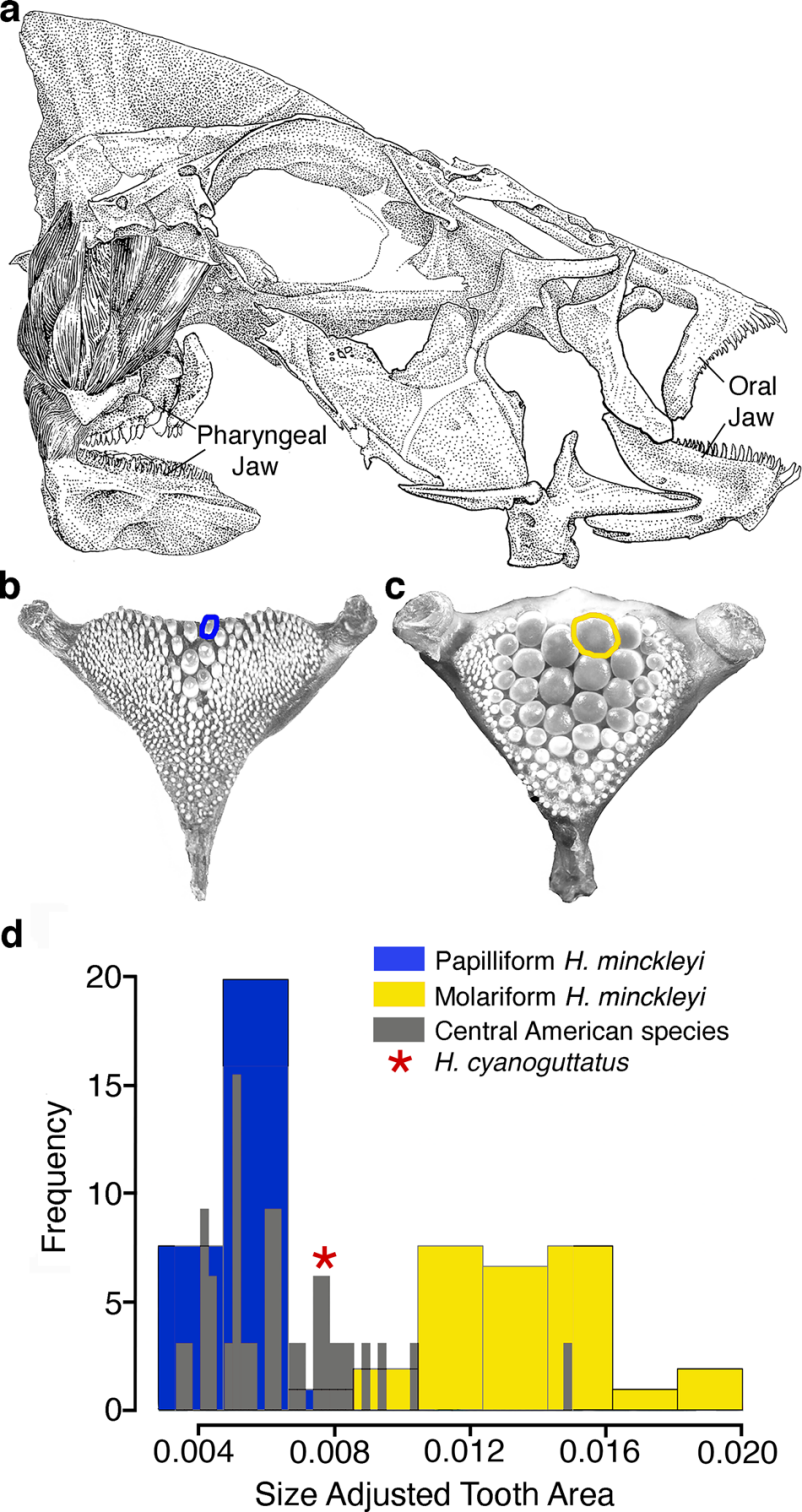
Intraspecific macroevolutionary divergence in *Herichthys minckleyi* tooth size. **a** Anatomical drawing of the cranial anatomy of *H. minckleyi* including its pharyngeal and oral jaws in a lateral view. The variation in *H. minckleyi*’s pharyngeal jaw and its distinct tooth morphologies is highlighted by a small toothed papilliform jaw, **b** and a large toothed molariform jaw, **c** The most posterior tooth along the right center line of the pharyngeal jaw was measured in 27 molariform and 29 papilliform *H. minckleyi*. **d** This pharyngeal tooth area was similarly measured in 34 Heroine cichlid species from Central America that are widely believed to have formed an adaptive radiation following their invasion of Central America. The variance in the pharyngeal tooth area of *H. minckleyi* rivals the phenotypic disparity seen in this much older ( > 15 million years) cichlid fish adaptive radiation.

Like most bony fishes, *H. minckleyi* and other cichlids possesses two toothed jaws (Fig. 1). Cichlids have oral jaws that are homologous to mammalian jaws and that are primarily used to capture prey^15^. Additionally, these fishes, possess toothed pharyngeal jaws that are modified gill arches used to process prey^16–18^. However, unlike most fish, *Herichthys minckleyi* is bimodal in its pharyngeal dentition^19–22^. Pharyngeal tooth area that can be easily measured from the flattened dorsal view readily divides *H. minckleyi* into one of two discreet morphotypes (Fig. 1). “Molariforms” have enlarged molar-like teeth on their pharyngeal jaws that aid in crushing their snail prey, and “papilliforms” exhibit only small, pointed teeth that increase their ability to shred aquatic plants^23,24^. This pharyngeal jaw variation in *H. minckleyi* is also associated with substantial differences in feeding ecology and diet^25,26^. The disparity in *H. minckleyi* tooth area has led to assertions that this fish’s dental polymorphism represents a case of “intraspecific macroevolution”^27^. However, whether the tooth variation approaches the macroevolutionary phenotypic divergence observable in more ancient and species diverse adaptive radiations like those in the African Great Lakes or the Central American Heroine cichlids that *H. minckleyi* is phylogenetically nested within remains unclear.

The discrete phenotypic divergence found in *H. minckleyi* might readily lead to inferences that its morphotypes have not only substantially evolved along an ecological axis but that they should be diagnosed as two distinct species^28–31^. However, the two pharyngeal morphotypes consistently occur together in sympatry and have been observed to interbreed readily in the wild in multiple geographically distinct locations^29^. The two morphotypes have also repeatedly been found to be genetically indistinguishable using a range of molecular markers including allozymes^24,29^, mitochondria^4^, microsatellites^32^, and thousands of restriction site associated single nucleotide polymorphisms (SNPs)^33^. This suggests either that the differences observed in *H. minckleyi* tooth size are entirely environmentally plastic^34,35^ or that the genetic underpinnings likely only involve a few, or perhaps even a single, genomic locus. The generation of a high-quality and annotated reference genome could facilitate a framework for the genomic dissection of *H. minckleyi*’s dental polymorphism^3,36^. Furthermore, a reference genome would provide the means to anchor QTL (quantitative trait loci) analyses to give insight into not only the location but the number of genomic regions responsible for pharyngeal differentiation in *H. minckleyi*^37,38^. Additionally, genome resequencing of individuals differing in their pharyngeal dentition would make it feasible to narrowly define and identify the genomic homology of loci underlying this pharyngeal differentiation^3,39^. A genome-wide analysis could greatly improve the fine-scale understanding of the genomic basis of this extensive dental divergence in the unique desert valley where *Herichthys minckleyi* is endemic.

**Fig. 2:**
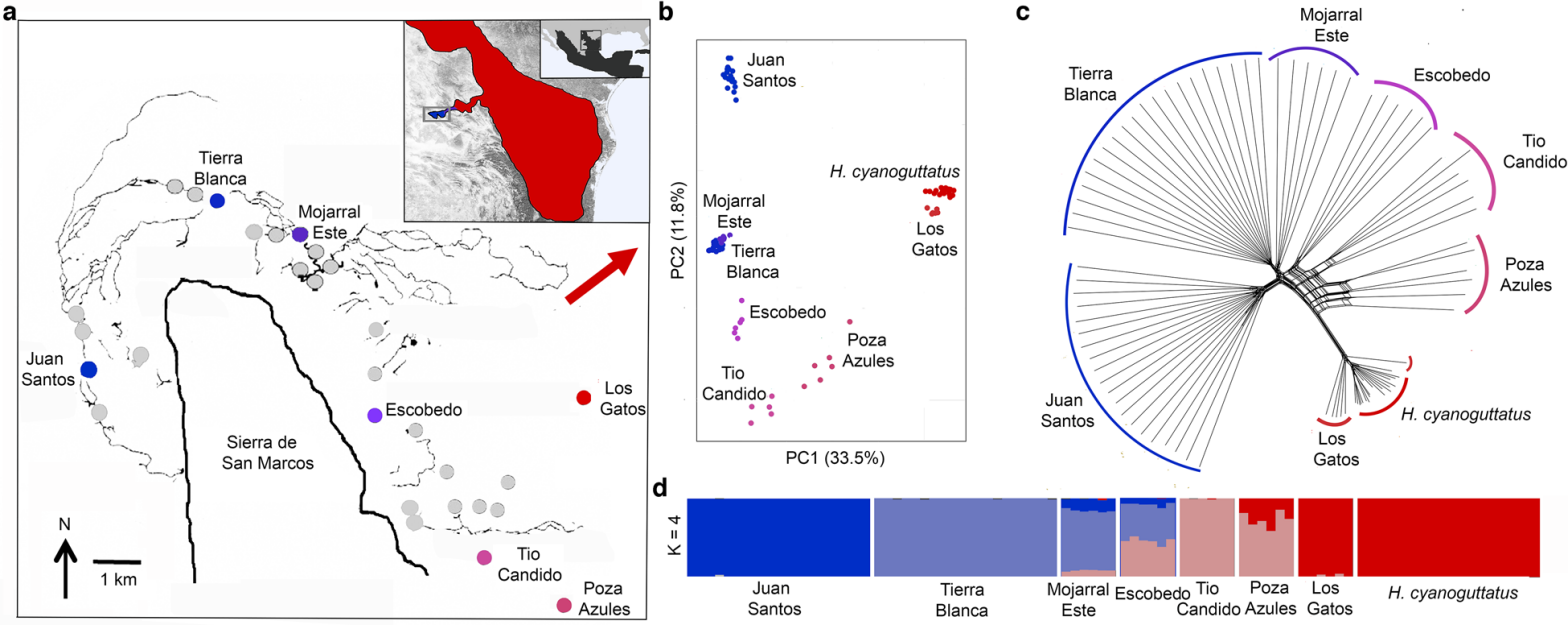
Population genomic and geographic divergence of *H. minckleyi* and *H. cyanoguttatus.* **a** Genomic samples of *H. minckleyi* (*n* = 70) and *H. cyanoguttatus* (*n* = 20) were taken across various locations in the small Cuatro Ciénegas valley and across the range of *H. cyanoguttatus* outside the valley (red shown in geographic inset with the range of cichlids in Mexico shown in black as further inset). The red arrow in the Cuatro Ciénegas map points to the nearest drainage where *H. cyanoguttatus* is native outside the valley. Morphotype designations and lab-based pharyngeal areas were phenotyped and genomes resequenced for molariform (M) and papilliform (P) *H. minckleyi* from the spring-fed pools of Juan Santos (M = 10; *P* = 10), Tierra Blanca (M = 10; *P* = 10), Mojarral Este (M = 2; *P* = 3), Escobedo (M = 3; *P* = 3), and Tio Candido (M = 2; *P* = 3). Individuals from Los Gatos (*n* = 6) and Poza Azules (*n* = 6) were sampled without taking vouchers for phenotyping. Although both pharyngeal morphotypes were present in all phenotyped populations, genome composition in the spring populations ranged from being more distinctly *H. minckleyi* (blue) to virtually identical to *H. cyanoguttatus* (red) genome sequences (n = 20) sampled from outside of the valley. **b** Genome-wide SNP divergence in *H. minckleyi* clearly grouped individuals by population as shown by PCA. **c** A genome-wide SNP distance network of the populations similarly highlighted the genetic distinctiveness of each the populations and the general cline of increasing similarity of southeastern Cuatro Ciénegas populations to *H. cyanoguttatus* sampled from outside the valley. **d** Admixture analyses supported distinct clusters (k = 4) that identified groups that transgressed some of the populations sampled but did not group by pharyngeal jaw morphology. There appears to be substantial introgression from *H. cyanoguttatus* into *H. minckleyi* in the Los Gatos and Poza Azules locations. However, consistent with previous studies of reduced representation SNPs^33,47^, the Tierra Blanca and Juan Santos populations showed limited genome-wide evidence of introgression of *H. cyanoguttatus* alleles.

The ecology and geography of *Herichthys* lineages in Northeastern Mexico provides an exceptionally tractable setting to test the mechanistic basis of phenotypic divergence with gene flow (Fig. 2a). Whereas many cichlid assemblages co-occur natively in massive tropical lakes or rivers^10,40,41^, *H. minckleyi* is the only cichlid fish endemic to a small ( ~ 40 × 40 km) valley called Cuatro Ciénegas located in the center of the Mexican Chihuahuan desert^42^. Within this valley, the pharyngeal morphotypes of *H. minckleyi* are commonly found together in a series of approximately 200 spring-fed streams and pools embedded within a dry desert matrix ringed by mountains^43,44^. Also, *H. minckleyi* and its close relative *H. cyanoguttatus* are the two northern-most cichlid species in the Neotropics^45^ and the range of *H. cyanoguttatus* currently overlaps *H. minckleyi* in a small lobe of the Cuatro Ciénegas basin^46^. Recent and extensive gene flow of *H. cyanoguttatus* into *H. minckleyi* is therefore geographically feasible and mitochondrial introgression between these two species has occurred^4,47^. However, what remains unclear is the genomic extent and morphological consequences of this introgression across the geographic range of the polymorphic *H. minckleyi*.

As has been observed in the initial stages of several adaptive radiations^8,48^ introgression from *H. cyanoguttatus* could have played a role in the eco-morphological divergence in *H. minckleyi*. Introgression could have substantially increased genomic variability in *H. minckleyi* across all the small and isolated populations in its geographically restricted range^49,50^. Hybridization could have also generated phenotypic novelty through transgressive segregation that produces recombinant phenotypes that “transgress”, or fall outside, the phenotypic distribution of *H. cyanoguttatus* and *H. minckleyi* hybrids^11,14^. Although introgression could simply introduce minor phenotypic variation^10,49^, hybridization could also lead to the introduction of loci that virtually instantaneously produce extensive phenotypic variation in a species even in the face of extensive gene flow^3,7^. Yet, if in a relatively simple system like Cuatro Ciénegas introgression can be refuted as the mechanism leading to phenotypic divergence, the rejection of this hypothesis would help bolster the idea that the extensive trophic diversity arose within the very small geographic range of the narrowly endemic *H. minckleyi*.

The extreme phenotypic divergence that segregates within *H. minckleyi* populations seems to have originated effectively instantaneously on an evolutionary timescale. This type of punctuated phenotypic divergence has largely been inferred only from the fossil record or for more qualitative traits like blindness, metamorphosis, or color patterns^51–54^. Punctuated change within populations that rivals the divergence seen during macroevolutionary diversification would be especially important to adaptive radiations more generally if it could be documented to have occurred in more quantitative and ecologically relevant phenotypes like tooth size. Critically, all vertebrate teeth, ranging from those in *H. minckleyi’s* pharyngeal jaws to human oral jaws, are homologous and derived from mineralized structures present in a common vertebrate ancestor^55–57^. Therefore, teeth provide an excellent organ system for determining how multiple levels of biological complexity have comparatively contributed to vertebrate diversification^58,59^. For instance, tooth size is one of the most frequently used traits to make inferences concerning macroevolutionary diversification in traits like body size, ecology, and species diversity^28,60^. Additionally, the astonishing diversity in form, arrangement, and regenerative capacity of vertebrate dentitions is largely produced through developmental mechanisms that are often surprisingly highly conserved^61–64^. However, despite our continually expanding knowledge of the developmental genetic mechanisms producing teeth and the clear importance of dentition to ecological divergence, few genes have been directly implicated in the adaptive divergence of vertebrate tooth morphology^65^.

Using a suite of genomic resources, we asked several questions about the tooth size polymorphism in *H. minckleyi*. First, to put the intraspecific divergence within *H. minckleyi* into a more macroevolutionary context, we asked how the phenotypic divergence in tooth size in this one cichlid species compares to divergence found in the Central American Heroine cichlid adaptive radiation. We also examined the geographic structure of population subdivision and gene flow from *H. cyanoguttatus* within the Cuatro Ciénegas valley where *H. minckleyi* is endemic. Then, we evaluated whether introgression from *H. cyanoguttatus* underlies the exceptional within population variation in *H. minckleyi* tooth morphology and could have contributed to the apparent punctuated intraspecific macroevolution and trophic based adaptive radiation within *H. minckleyi*. Finally, we asked what the genomic architecture and individual loci might be that are responsible for the dental polymorphism in *H. minckleyi*.

## Results

### Tooth area comparative analyses

There was substantial variation in both *H. minckleyi* (Fig. 1) and other Central American cichlids (Supplementary Tables 1,2). The coefficient of variation (CV) of size-adjusted tooth areas in species means for the 33 Central American cichlid species was 0.184. For the 56 *H. minckleyi*, the value was 0.287. In *H. minckleyi*, the intraspecific variation as measured by CV of pharyngeal tooth size was significantly higher (*P* = 0.002) than the interspecific variation in the mean values of the remaining Central American cichlid radiation.

### Herichthys cyanoguttatus reference genome

Several elements contributed to the assembly of the *Herichthys cyanoguttatus* reference genome that shows broad similarity to many other high quality cichlid reference genomes (Supplementary Tables 3,4). The average N50 of our long-read sequencing subreads was 18.1 kilobases (kb), resulting in a total of 72 gigabases (Gb) ( ~ 81x coverage) for our genomic backbone sequence data. A total of 147 Consensus physical maps (CMAPs) with an N50 of 30.95 megabases (Mb) and total CMAP length of 1072.96 Mb were then generated. Hi-C reads totaling 352 million Illumina paired-end 101 bp reads at ~40x coverage were used to further polish and scaffold the genome. The assembled genome comprised 93 total scaffolds that sum up to 889,498,433 bp. Importantly, the polished scaffolding resulted in 24 super scaffolds that included 98.2% of the assembled genome, and these super scaffolds corresponded to the expected chromosome number for the genus *Herichthys*^66^. Annotation of the genome produced a gene set that included 25,769 protein-coding genes and 28,343 transcripts.

### Genetic structure

All analyses of population genomic structure suggested that geography explained a substantial degree of the genome-wide divergence in *H. minckleyi* (Fig. 2; Table 1). These inferences were all based on our variant filtering scheme that yielded a variant call format (VCF) file containing 870951 SNPs. Pruning based on linkage disequilibrium (LD) reduced the size of our dataset for population genomic analyses to 137,703 SNPs. Whether we examined these SNPs in a genome-wide PCA, distance-based neighbor-network, or admixture analyses, the *H. minckleyi* samples formed distinct clusters in accordance with their geography (Supplementary Table 5). More western populations (Juan Santos, Tierra Blanca, and Mojarral Este) grouped apart from the more eastern populations (Escobedo, Tio Candido, and Poza Azules) along the first PC axis.

**Table 1:** *Herichthys* population divergence

Mean genome-wide *Fst* of sampled locations down-sampled to 6 individuals (*n* = 3 molariform and *n* = 3 papilliform *H. minckleyi* when phenotyped) per population is shown above the diagonal. The red coloration highlights the low *Fst* indicative of extensive gene flow between Los Gatos and *H. cyanoguttatus*. Blue highlights degrees of population subdivision with darker blue in a cell representing higher *Fst* than light blue and white. The linear geographic distances calculated from GPS coordinates between *H. minckleyi* sampled populations are given below the diagonal in kilometers (km). Darker gray represents greater distances. Native *H. cyanoguttatus* populations used in this analysis were sampled from the Rio Salado, a tributary of the Rio Grande, approximately 40 km outside of the Cuatro Ciénegas valley. There is substantial genome wide differentiation in *H. minckleyi* over relatively small ( < 10 km) geographic distances.

Comparison of the different *H. minckleyi* populations revealed patterns characteristic of some gene flow with *H. cyanoguttatus* that was also geographically structured (Fig. 2). The six *H. minckleyi* collected from Los Gatos cluster closely with *H. cyanoguttatus* both in the neighbor-network and genome-wide PCA. The individuals from Poza Azules and Tio Candido also cluster in the neighbor-network apart from *H. minckleyi* of the more western localities and more toward the cluster comprised of *H. cyanoguttatus* and Los Gatos *H. minckleyi*. The genome-wide mean Weir and Cockerham *F*_*ST*_ estimate obtained comparing Los Gatos *H. minckleyi* and *H. cyanoguttatus* is also very low ( ~ 0.066) compared to those measured for other populations with *H. cyanoguttatus* (Table 1). The next lowest being Tierra Blanca with a mean *F*_*ST*_ of ~0.201. Admixture analysis also suggested substantial shared ancestry between some *H. minckleyi* populations and *H. cyanoguttatus*. The individuals collected from Los Gatos showed the strongest patterns of admixture with *H. cyanoguttatus*. The admixture analyses were run for a range of population subdivisions, with 4 populations showing the lowest cross-validation (CV) error (Supplementary Fig. 1). Assuming four populations (*K* = 4), more than 90% of the genomic contribution of the six Los Gatos individuals is assigned to the same group as *H. cyanoguttatus*. Furthermore, roughly a third of each Poza Azules individual’s genome shared the same assignment as *H. cyanoguttatus*. The Escobedo and Mojarral Este individuals all exhibited varying degrees of admixture of the three distinct ancestral groups assigned to the Tio Candido, Tierra Blanca, and Juan Santos populations. While each of the population genomic analyses recovered a strong phylogeographic signal, there appears to be minimal to no structuring to the genomic divergence based on the *H. minckleyi* pharyngeal morphotype. Within the Mojarral Este (*F*_*ST*_ < 0.000), Juan Santos (*F*_*ST*_ = 0.003), Tierra Blanca (*F*_*ST*_ < 0.000), Escobedo (*F*_*ST*_ < 0.000) and Tio Candido (*F*_*ST*_ = 0.009) populations, the mean genome-wide *F*_*ST*_ for their molariform and papilliform *H. minckleyi* was consistently estimated to be negligible, especially compared to the substantial geographically associated among population divergence.

### QTL analyses

**Fig. 3:**
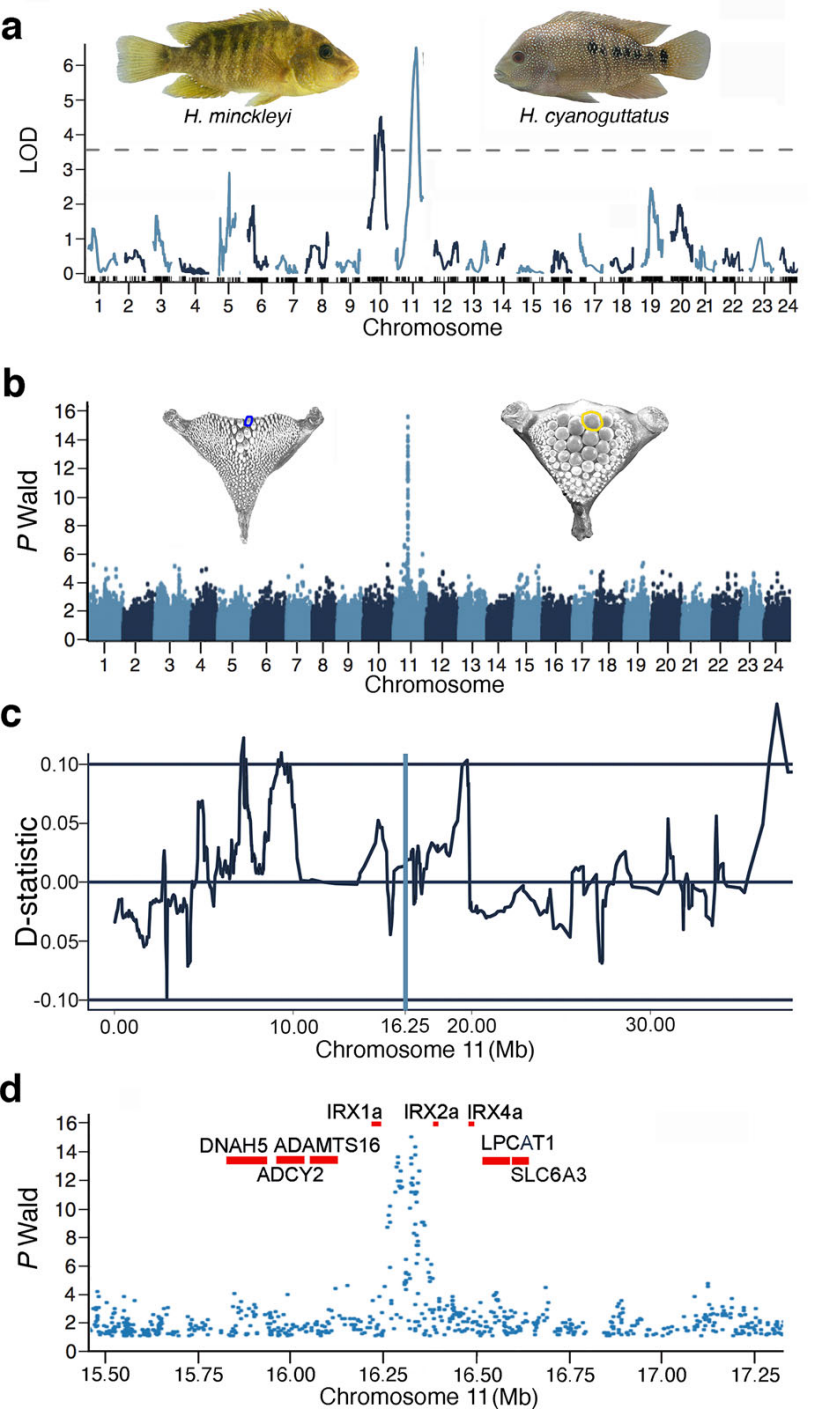
Genomic basis of *H. minckleyi* tooth area. **a** The single largest effect QTL was identified in the 198 F2 hybrid mapping panel of the *H. minckleyi* x *H. cyanoguttatus* genetic cross to be located on chromosome 11. **b** Based on genome resequencing in the 56 phenotyped *H. minckleyi* (M = 27; *P* = 29) constituting the wild-caught mapping population, the *H. minckleyi* pharyngeal tooth polymorphism showed a single SNP peak of high association on chromosome 11. **c** Dsuite sliding window analysis of chromosome 11, depicted here with a 100 SNP sliding window and 20 SNP step size, showing no evidence of introgression around the peak of association (blue line). **d** A zoomed in view of this peak of association highlights that the most highly associated SNPs mapped to the intergenic region between *IRX1a* and *IRX2a* in the newly sequenced and annotated *H. cyanoguttatus* genome.

The F2 linkage map was blanketed with 3087 ddRAD generated SNP markers distributed across 24 linkage groups (Supplementary data 1). The variation in the F2 tooth areas approximated a normal distribution that spanned the range from an average papilliform *H. minckleyi* and the average tooth size of an *H. cyanoguttatus* (Supplementary Fig. 2). This variation was not consistent with extensive transgressive segregation of tooth types and was noticeably not bimodal. However, from this *H. minckleyi* x *H. cyanoguttatus* F2 hybrid cross (Fig. 3), we inferred there were four QTL regions that were found to be significant at the chromosome level and together they explained 37% of the variation in tooth area (Supplementary Table 6). Two of the peaks of association on chromosome 5 and 19 explained approximately 10% of the variation each. Another peak on chromosome 10 explained over 10% of the variation in tooth area. However, the highest peak of association, explaining over 19% of the variation in tooth area in the F2 mapping population, was located within a region ranging from 12.64 - 26.24 Mb on chromosome 11.

### Interspecific gene flow D-statistics

By quantifying D-statistics in sliding windows and leveraging the *H. cyanoguttatus* genome sequence, we identified several genomic regions that showed evidence of elevated gene flow from *H. cyanoguttatus* into one of the *H. minckleyi* morphotypes. However, when we examined the D-statistics on chromosome 11 that contained the largest tooth area QTL and the GWA peak of association (see below), we recovered limited evidence of putatively introgressed alleles from *H. cyanoguttatus* (Fig. 3). Critically, and despite the use of a range of genomic windows, these results did not support the hypothesis that introgression was elevated in the region containing the single locus underlying the polymorphism that we identified below on Chromosome 11 through genome wide association mapping.

### Genome wide association mapping

Chromosome 11 carried a substantial number of SNP variants that fell within a single peak of association (Fig. 3) and showed high association with the polymorphism in *H. minckleyi* (Table 2). The five most significantly associated SNPs all fell within a 50 kilobase (kb) window and were highly associated with *H. minckleyi* tooth area (*P Wald* < 1e-13.5) (Supplementary data 2). This single peak of association was the only clearly identifiable GWAS peak separating the two *H. minckleyi* morphotypes. This significant peak was located in non-coding regions between the *IRX1a* and *IRX2a* genes that form a highly conserved gene cluster with the *IRX4a* gene (Fig. 3c). The five most highly associated SNPs with the polymorphism were found within a single ~46 kb window (Table 2). For these SNPs, *H. cyanoguttatus* was consistently fixed homozygous for the reference allele. In contrast, the molariform *H. minckleyi* even if they were considered their own isolated population showed a substantial excess of heterozygosity, with one “*H. minckleyi*” allele and one *H. cyanoguttatus* allele being by far the most common genotype. For these five SNPs, the *P* value ranged from 0.012 to 0.034 and Cramer’s V effect sizes ranged from medium to large, 0.407 to 0.483. In contrast to the molariforms, the papilliform individuals at three of these physically co-located SNPs were fixed homozygous for the alternative “*H. minckleyi”* alleles.

**Table 2:** Top five SNPs most highly associated with the *H. minckleyi* pharyngeal jaw tooth polymorphism

Genomic	R	A	*P-Wald*	*P-score*	*H. cyanoguttatus*	*H. minckleyi*		HWE	
Location						P	M	*P*	*V*
11:16290331	G	A	13.684	1.18E–07	11/0/0	0/0/28	4/20/3	0.012	0.483
11:16325081	A	T	15.245	5.75E–08	12/0/0	0/0/29	4/20/3	0.012	0.483
11:16325588	C	T	13.662	1.16E–07	9/0/0	0/0/28	5/20/3	0.021	0.436
11:16334484	T	C	13.903	1.06E–07	14/0/0	27/0/0	4/19/4	0.034	0.407
11:16335817	A	G	14.51	8.05E–08	15/0/0	29/0/0	4/19/4	0.034	0.407

These SNPs were located within a ~ 50 kb genomic interval on chromosome 11 between the genes *IRX1a* and *IRX2a*. The reference allele (R) from the *H. cyanoguttatus* genome and alternative (A) allele are shown for each SNP. The *P-Wald* and *P-score* for the association with tooth size with each SNP are given. Then, the number of homozygous reference/heterozygote/homozygous genotypes for *H. cyanoguttatus*, papilliform (P) *H. minckleyi*, and molariform (M) *H. minckleyi* are shown. Strikingly, *H. cyanoguttatus* are fixed for all loci as are papilliform *H. minckleyi*. However, despite the genomic distance, the papilliform *H. minckleyi* are fixed for the alternative allele in the first three and the reference allele for last two. Based on Hardy-Weinberg expectations (HWE) if they were their own population, a chi-square test suggests the molariform *H. minckleyi* genotypes exhibit significant (*P*) excess heterozygosity with medium to large effect size (*V*).

## Discussion

The genomic basis of the intraspecific trophic polymorphism in *H. minckleyi* suggests changes in a single, small genomic region can favor virtually instantaneous macroevolutionary divergence. The variation in this polymorphic species rivals the eco-phenotypic dental divergence present in the much older and species rich cichlid adaptive radiation in Central America (Fig. 1c). The proportion of the *H. cyanoguttatus* genome in cichlid populations within Cuatro Ciénegas was determined to range from nearly complete to minimally detectable within *H. minckleyi* exhibiting both morphotypes. Our laboratory QTL analyses indicate hybridization can occur between the two species and that hybrids can produce heritable variability in tooth size. However, this variation was not bimodal and did not generate the full range of tooth area seen in *H. minckleyi*. Leveraging the newly generated and annotated *H. cyanoguttatus* reference genome to anchor our *Herichthys* hybrid F2 mapping population and the population genomic resequencing, indicated the *H. minckleyi* dental polymorphism is inferred to have been generated primarily by divergence in a single non-coding region nested between the genes *IRX1a* and *IRX2a*. The alternative fixation of SNP alleles in this region and the excess heterozygosity observed in molariform *H. minckleyi* argue that introgression at this locus could have promoted a Menedelian basis to this dental polymorphism. The genomic basis of *H. minckleyi*’s extreme variation in tooth size blurs the lines between micro- and macroevolution.

Despite the lack of population genomic structuring based on *H. minckleyi*’s pharyngeal morphotype, there is strong phylogeographic signal present in the re-sequenced genomes (Fig. 2). The cichlids sampled from different springs across the Cuatro Ciénegas basin show extensive genomic differentiation associated with geography (Fig. 2, Table 1). Substantial geographic structuring among *H. minckleyi* populations is also consistent with previous molecular phylogeographic analyses of *H. minckleyi* and several other aquatic species in the Cuatro Ciénegas valley^43,44,50,67^. The desert matrix in which the genome-wide population structuring of spring-dwelling *H. minckleyi* occurs likely provides a frequent but not a complete barrier to gene flow.

The different *H. minckleyi* populations were inferred to exhibit gene flow to varying degrees with *H. cyanoguttatus* (Fig. 2). Previous population genomic studies have provided a range of inferences regarding Cuatro Ciénegas gene flow as well as the extent of introgression of *H. cyanoguttatus* into *H. minckleyi*^4,24,32,33^. However, the amount of gene flow from *H. cyanoguttatus* appears to be most substantial in the southeastern lobe of the valley at locations such as Los Gatos and Poza Azules. In contrast, there is relatively little trace of the *H. cyanoguttatus* genome in the more northern and western locations sampled. As has been suggested for other species in Cuatro Ciénegas^43,44^, the human made canals that around 100 years ago connected the aquatic habitats of this previously isolated valley with Rio Grande drainages likely provided the conduit for *H. cyanoguttatus* invasion and the introgression documented here^47^. Surprisingly, the Los Gatos populations within Cuatro Cienegas were more genomically similar, as indicated by lower *Fst*, to the *H. cyanogutatus* sampled from outside of the Cuatro Cienegas valley than to the other cichlid populations within the valley (Table 1). Importantly, the geographic structuring present in *H. minckleyi*’s population genomic differentiation is therefore not due primarily to differential introgression from *H. cyanoguttatus*.

The genomic dissection of tooth size variation in *Herichthys* was further investigated through both QTL analyses and genome-wide association of SNPs. The QTL analyses indicated that more than one locus could be responsible for tooth size differences between *H. cyanoguttatus* and *H. minckleyi* (Fig. 3a). In contrast, the genome-wide results suggest a single locus that overlaps with the largest QTL identified is likely responsible for tooth size variation in *H. minckleyi* (Fig. 3b). This minor discrepancy could have multiple explanations. The causal locus could have distinct phenotypic effects, or penetrance, in the different genomic backgrounds of the two *Herichthys* species^11,12^. Also, there could be many modifier loci or additional mutations in the genome of one species that cause a single locus of large effect to exhibit less phenotypic consequence in another species^3^. The slightly larger number of loci detected in the QTL cross could therefore reflect additional modifier loci in the genome of *H. cyanoguttatus* for tooth size^39,54^. Further, *H. cyanoguttatus* and *H. minckleyi* are estimated to have shared a common ancestor several million years ago^22^. This timeframe provides ample opportunities for mutations to accumulate in both the entire genome and even within the IRX cluster of the two cichlid species that could have produced substantially different tooth size phenotypes^36,37^. Regardless, both genetic approaches supported a locus on chromosome 11 as having a large phenotypic effect on tooth size.

The simple genomic basis that was elucidated through our combined as well as overlapping QTL and whole genome results (Fig. 3) helps to explain the bimodality of *H. minckleyi* tooth area (Fig. 1). Despite tooth area often being considered a quantitative trait, this morphotype-determining locus likely acts similarly to many classic Mendelian loci in producing effectively qualitative differences in *H. minckleyi*’s pharyngeal tooth phenotype (Fig. 1c)^2,68^. These genome-wide results also help us to re-interpret, and importantly do not contradict, the numerous studies that have previously employed genetic markers to investigate the *H. minckleyi* polymorphism^4,19,20,24,32,33,47^. These previous studies all analyzed relatively small subsets of the standing genomic variation and found no genetic differentiation between the two *H. minckleyi* pharyngeal morphotypes. For almost all of the genome, we also recovered a pattern consistent with genomic homogenization for the two pharyngeal tooth morphotypes (Figs. 2,3). This lack of genetic differentiation between the morphotypes across virtually the entire genome is consistent with little, to no, assortative mating based on pharyngeal morphotypes as well as the documented interbreeding of the *H. minckleyi* pharyngeal morphotypes in the wild^29^. This inbreeding has been observed at four different Cuatro Ciénegas spring localities. Further, at the most intensively studied field location, over 50% of the 33 observations of mating pairs were found to occur between individuals with different pharyngeal morphologies. The internal pharyngeal jaw tooth variation in *H. minckleyi* is clearly not associated with complete reproductive isolation and has not promoted genome-wide differentiation.

Our genome-informed analyses of gene flow found no evidence that introgression from *H. cyanoguttatus* was associated with the single locus found to be associated with the dental polymorphism (Fig. 3). A number of studies have found evidence of hybridization early on during adaptive radiations and many of the most diverse cichlid radiations show evidence of hybridizing gene flow during the initial stages of diversification^48,69^. However, most cichlid assemblages contain many recently diverged and currently sympatric species that long after speciation remain capable of hybridizing^9,10,70–72^. Also, as extinction does happen and distributions do change, incomplete lineage sorting, shared ancestral polymorphism, or introgression from a third unexamined species could always be a source of within species variation^8,39^. But, testing whether the effects of introgressive hybridization on adaptation and speciation in many of these systems remains somewhat difficult^14,49^. In contrast, the simplicity of the *H. minckleyi* system in Cuatro Ciénegas could provide a relatively tractable system for determining what role that *H. cyanoguttatus* introgression has played in generating diversity in other phenotypes present in *H. minckleyi* (Fig. 3). Nevertheless, we did not find evidence that introgression from *H. cyanogutatus* generated the genetic variation underlying the pharyngeal polymorphism in *H. minckleyi*.

The *H. minckleyi* pharyngeal morphotypes are extensively differentiated at one locus centered around a cluster of three *IRX* genes. The genomic mapping of the tooth morphology supports the idea that gene clusters like the *IRX* cluster implicated here could often play an outsized role in the evolution of phenotypic novelty^60,73^. *IRX* genes have previously been found to play a central role in the development of teeth in several vertebrates^73,74^. For instance, *IRX1* is crucial for dental epithelial cell proliferation, differentiation, and influences the continued incisor growth in mice^75^. Consistent with what has been found in other fishes and here in the *H. cyanoguttatus* genome, vertebrate *IRX* genes often exhibit conserved clusters of three genes. The *IRX* cluster and the surrounding genes that map to the region provide a robust basis to homologize the *IRX1a*, *IRX2a*, and *IRX4a* cluster to other cichlids and additional teleosts^3,37^. Based on their expression patterns in other vertebrates, *IRX* genes very likely regulate morphogenesis in multiple tissue types and future studies should examine if this could explain the differentiation in other traits like the musculature and digestive tract that also characterize the distinct pharyngeal dental morphotypes in *H. minckleyi*^22,23,27,31,76^. However, addressing specific *IRX* gene function is difficult because of their genetic redundancy and shared regulatory elements. Within and between clusters, *IRX* genes display similar and overlapping expression patterns, explained in part by super-enhancers that co-regulate the gene expression of *IRX* genes within a cluster^77^. Therefore, changes in the non-coding regions of this cluster where the most highly associated SNPs in *H. minckleyi* are found could directly influence expression of multiple *IRX* genes as well as their known downstream targets. Genomic change at highly conserved and developmentally critical loci like this *IRX* cluster that can influence multiple phenotypically pleiotropic and ecologically important traits could frequently generate an outsized component of the substantial morphological variation found in adaptive radiations at the earliest stages of morphological divergence.

The nature of the genomic variation at the *IRX* locus in *H. minckleyi* might not only be responsible for the origin of the tooth variation but also the maintenance of this polymorphism (Fig. 3). First, because there would be no issue with recombination breaking up multiple causative loci, the indication that a single locus is responsible for the tooth size variation makes the maintenance of this polymorphism in the face of gene flow more tractable^3,78^. Additionally, the papilliform individuals in *H. minckleyi* are often homozygous in this region for “*H. minckleyi”* alleles wherein the molariform *H. minckleyi* are heterozygous (Table 2). The molariform commonly exhibit one *H. minckleyi* allele and one allele that is fixed in *H. cyanoguttatus* populations. This pattern of differentiation suggests there could be some kind of inversion or some other structural variation in the genome that governs these frequencies of discrete mixtures of *H. cyanoguttatus* alleles in the presence of a *H. minckleyi* genomic background^79,80^. Future studies using longer genomic reads from *H. minckleyi* should detail whether genomic rearrangements or other structural modifications are playing a central role in maintaining *H. minckleyi*’s pharyngeal tooth size diversity.

Adaptive processes have also likely favored the origin and maintenance of the genomic variation underlying *H. minckleyi’s* dental variability. The two pharyngeal morphotypes in *H. minckleyi* are quite trophically differentiated. This ecological divergence in the two morphotypes has been shown to reduce competition in the wild, suggesting that the resulting intraspecific phenotypic diversification could be favored by disruptive selection^25^. Functionally, the papilliform pharyngeal morphotype is specialized to shred plant material and the molariform morphotype is exceedingly well-suited to crushing several of the snail species that are endemic to Cuatro Ciénegas^22,23,26^. Although dental plasticity induced from crushing snails might in part be responsible for the tooth size differentiation in this cichlid, our genetic results argue the variation in the polymorphism is not simply due to environmentally induced phenotypic plasticity^34,35^. However, the genomic basis of the polymorphism and the environment in Cuatro Ciénegas have likely interacted to favor and maintain the adaptive trophic variation in *H. minckleyi*.

Tooth size diversity in this one species rivals a substantial amount of the diversity found in a much older and species rich assemblage of Central American cichlids (Fig. 1c)^40,45^. Therefore, *H. minckleyi* provides an extreme empirical example of how even within a small geographic range and limited timeframe over which substantial diversification in adaptive radiations can proceed. Even at the initial stages of diversification within a single species, a burst of phenotypic diversity can originate that might almost immediately produce the maximum phenotypic disparity found in a clade. Macroevolution as a discipline will continue to focus primarily on the substantial phenotypic and genomic diversity that accumulates in clades of organisms after they diversify into multiple species^70–72,81,82^. However, even at the earliest stages of adaptive radiation within populations, isolated genomic changes that are geographically localized and genetically simple can produce macroevolutionary-like intraspecific phenotypic diversity.

## Methods

### Field collections

Specimens of *Herichthys minckleyi* and *H. cyanoguttatus* were collected from the field using standard methods at various time points between 2000 and 2010 (Supplementary Table 1). All collections followed protocols approved through the country of Mexico and the University of Tennessee’s Institutional Animal Care and Use Committee (IACUC). We have complied with all relevant ethical regulations for animal use. In the field, it is generally possible to unambiguously assign *H. minckleyi* to either the molariform (M) or papilliform (P) morphotype (Fig. 1b, c)^30,31^. Following exposure to tricaine methane sulfonate (MS-222), field-based pharyngeal *H. minckleyi* phenotypes were assessed using an otoscope placed into the oral jaws of the fish. If enlarged molariform teeth were present, the individual was classified as molariform. If only small and pointed teeth were present on the pharyngeal jaw, the individuals were classified as papilliform. Small individuals ( < 70 mm) and the relatively rare ( < 5%) *H. minckleyi* with difficult to diagnose pharyngeal morphology were not included in analyses. Within the Cuatro Ciénegas valley, *H. minckleyi* were collected, phenotyped, and individually tagged from the spring-fed pools of Juan Santos (M = 10; *P* = 10), Tierra Blanca (M = 10; P = 10), Mojarral Este (M = 2; P = 3), Escobedo (M = 3; *P* = 3), and Tio Candido (M = 2; *P* = 3). Individuals from Los Gatos (*n* = 6), Mojarral Este (*n* = 1), and Poza Azules (*n* = 6) were also fin-clipped for sequencing but physical vouchers were not taken or phenotyped.

Quantitative tooth phenotypes of the tooth areas were also generated for the 27 molariform and 29 papilliform *H. minckleyi* used for subsequent genome-wide association and comparisons with other cichlids (Supplementary Table 1). To quantify pharyngeal tooth areas, we first dissected the lower pharyngeal jaws from the fishes. These bony elements were cleaned of all muscle and fascia and allowed to dry. Then, we took a digital image of the dorsal view of the jaws with a ruler in frame and imported it into the program ImageJ (v.1.51)^83^. Using this program, the dorsal area of the posterior-most tooth along the right of the suture that divides the pharyngeal jaw^31^ was digitally quantified. For all specimens, we also measured the standard length (SL), the distance from the upper jaw tip to the caudal peduncle, with dial callipers on each specimen to control for body size. To linearize and size-standardize the tooth area measurements, the ratio of the square-root-transformed tooth areas and standard length were calculated and then used for subsequent genetic and comparative analyses.

### Tooth area comparative analyses

To contextualize the phenotypic diversification of *H. minckleyi* within an evolutionarily older and widely recognized adaptive radiation^40,45^, we compared the phenotypic divergence in pharyngeal tooth area present in *H. minckleyi* to that found in 33 other species of Central American Heroine cichlids (Supplementary Table 2). These species were chosen based on availability but represent approximately 1/3 of the species as well as a robust trophic subsampling of this diverse cichlid radiation that dominates the fish fauna of Central America. The pharyngeal tooth areas were phenotyped with digital images as above for *H. minckleyi* following collection from their native range (Supplementary Table 1)^46,84^. Tooth areas of between 1 and 6 individuals per species were measured, size-standardized, and then averaged to produce species values for comparison with *H. minckleyi* intraspecific variation. The variation between the species size-adjusted mean tooth areas and *H. minckleyi* tooth areas were contrasted using the R package cvequality (v.0.2.0)^85^. Effectively, we compared whether the variances of size-adjusted mean tooth areas in *H. minckleyi* were significantly different from the variation seen in a substantial interspecific subsample of the trophically diverse Central American Heroine cichlid radiation.

### Reference genome assembly

A reference genome of *H. cyanoguttatus* was constructed to anchor our genomic analyses. Because of its previous evidence of hybridization with *H. minckleyi*, this species is less imperiled, and it is more tractable for generating genomic resources^64^ an *H. cyanoguttatus* individual was chosen as the genomic reference. To obtain high molecular weight genomic DNA for this cichlid, snap frozen liver tissue was extracted from a single *H. cyanoguttatus* individual collected from a small tributary of the Rio Grande, Texas, USA. Liver tissue was ground to a fine powder in liquid nitrogen. Powdered liver tissue was lysed overnight at 55 °C in high salt tissue lysis buffer (400 mM NaCl, 40 mM Tris pH 8.0, 30 mM EDTA, 0.5% SDS, 100 µg/µl Proteinase K). RNA was removed by incubation with 200 μg/ml RNaseA for 1 hour at 37°C. RNase A for 1 hour at 37°C. High molecular weight genomic DNA (HMW gDNA) was purified by three washes with phenol-chloroform-IAA equilibrated to pH 8.0, followed by two washes with chloroform-IAA, and precipitated in ice-cold 100% ethanol. Filamentous HMW gDNA was spooled and was washed twice with 70% ethanol, dried at 37 °C for 10 min and eluted in TE buffer. An additional post-purification step was performed to remove potential carbohydrate contaminants by adding 1/10 volume of 0.3 M NaAcetate to precipitate carbohydrates. The supernatant containing HMW gDNA was collected. The length of the HMW gDNA molecules was between 50 and 400 kb as determined by pulse field gel electrophoresis.

Subsequently, continuous long read (CLR) library preparation was performed according to the Pacific Biosciences (PacBio) ‘Guidelines for preparing size-selected 20 kb SMRTbell^TM^ templates’ using the PacBio Template Prep Kit 1.0. First, 20 µg of HMW gDNA was sheared using the Megaruptor^TM^ device (Diagenode) according to the manufacturer’s instructions to obtain fragment sizes of approximately 25 kb. Then, approximately 16 µg of sheared gDNA was used for library preparation. The PacBio SMRTbell^TM^ library was size selected for fragments larger than 18 kb using the BluePippin^TM^ device according to the manufacturer’s instructions. SMRT sequencing was performed on the SEQUEL system at the DRESDEN concept Genome Center (DcGC) in Dresden, Germany, using Sequencing Chemistries 2.0. The movie duration was 10 hours each for the successfully sequenced 21 SEQUEL SMRT cells.

Reads from the 21 PacBio SMRT cells were assembled using the *MARVEL* assembler^86,87^ with default parameters unless mentioned otherwise. *MARVEL* consists of three major steps, namely the setup phase, the patch phase, and the assembly phase. In the setup phase, reads were filtered by choosing only the longest read over a minimum read length of 13 kb. The resulting 2.1 million reads ( ~ 52x coverage) were stored in an internal database. The patch phase detects and corrects read artefacts including previously missed adapters, polymerase strand jumps, chimeric reads, and long low-quality read segments that are the primary impediments to long contiguous assemblies. The local alignment computation is by far the most time and storage consuming part of the pipeline. Therefore, a repeat masking strategy was applied that differs from the default *MARVEL* pipeline but can be more easily applied on computing clusters. Low complexity intervals, such as microsatellites or homopolymers, were annotated with *DBdust* (https://github.com/thegenemyers/DAZZ_DB) and tandem repeat elements were analyzed with *datander* and *TANmask* (included in *MARVEL* developmental branch). Furthermore, local alignments of 1x coverage against 1x coverage of the genome were computed with *daligner* (https://github.com/thegenemyers/DALIGNER) and then alignment piles of size 10 and greater were used to generate repeat interval tracks. The resulting repeat tracks (dust, tan, repeat) were subsequently used to compute all local alignments between all blocks of the database. The patched reads ( ~ 48x coverage) were then used for the final assembly phase beginning with determining all overlaps of patched reads. The previously created repeat annotation was reused and the trace spacing was set to 126 to force *daligner* to store the traces into a 16-bit buffer. This modification increases the storage demands on average by 20% but ensures the use of a modified version of *LAstitch*. This modified version stitches short alignment artefacts resulting from bad sequencing segments within overlapping read pairs to align through low complexity or tandem repeat elements without creating an overflow when using the default 8-bit compression of traces. This step was followed by a more precise repeat annotation and the generation of the overlap graph. The final contigs were generated by touring the overlap graph. Finally, to correct base errors, we first used the correction module of *MARVEL*, which makes use of the final overlap graph and corrects only reads that were used to build contigs. By using an alignment-based approach, the final contigs were further separated into a primary and an alternative contig set.

Optical mapping was performed using the Bionano Prep Direct Label and Stain DLS DNA Kit (Bionano Genomics) according to the manufacturer’s protocol. Briefly, 750 ng of ultra-long gDNA extracted from muscle was sequence-specifically labeled with a fluorophore-labeled nucleotide using the non-nicking Bionano Direct Label Enzyme (DLE-1). For further visualisation, the DLE-1 labeled gDNA backbone was stained with DL-Green. Labeled molecules were imaged using the Bionano Saphyr system at the DcGC in Dresden, Germany. Data were generated from Bionano flow cells and molecules larger than 150 kb were combined for a total yield of 320 Gb.

A genomic map was assembled de novo and used to order and to orient the contigs from the *MARVEL* PacBio assembly, and to correct remaining contig mis-assemblies. Consensus physical maps (CMAP) were assembled using *Bionano Solve 3.3_10252018* (https://bionanogenomics.com/support-page/bionano-solve/)^88^. Molecules were filtered for a minimum length of 150 kb and a minimum of 9 labels on each molecule (DLE1: *n* = 1,179,796; approximately 970GB raw 1090x coverage). A *P*value threshold for the optical mapping assembly was set to at least 1 × 10^–10^. A total of 147 CMAPs (N50 of 30.95 Mb; total CMAP length of 1072.96 Mb) was generated for use in the hybrid-scaffolding pipeline of *Bionano Solve 3.3_10252018* set with default parameters. The process of hybrid scaffolding includes alignment of the PacBio assembly to the Bionano physical maps, identifying and resolving conflicting alignments, merging of nonconflicting assembly and CMAPs into hybrid scaffolds, and then a final translation back to FASTA format.

To further order and orient scaffolds to chromosome scale, genome-wide chromatin interaction data (Hi-C reads) and SALSA2 were used^89^. Hi-C reads were sequenced at PhaseGenomics (in total 352 M Illumina paired-end 101 bp reads; ~40x coverage). As input for SALSA2, the Bionano hybrid scaffolds were used together with the *Arima mapping pipeline* (https://github.com/ArimaGenomics/mapping_pipeline). To reach an error rate of Q40, the final scaffolds were further polished. To do so, the whole data set of PacBio raw reads ( ~ 81x coverage) were mapped to the scaffolds and the consensus sequence was called with PacBio’s *Arrow* tool. To improve the performance, especially in repetitive regions, *Arrow* polishing was applied twice consecutively. To further correct base errors and reduce remaining length errors in homopolymer regions, 10x read clouds were used. To map 10x read clouds to the Arrow-polished scaffolds, the 10x Genomics Longranger align pipeline (https://github.com/10XGenomics/longranger, v.2.2.0) was applied, which uses the barcode-aware mapping tool Lariat^90^. Afterwards the variant detector FreeBayes (v.1.2.0)^91^ was used to identify polymorphic positions and bcftools consensus (v.1.9)^92^ (https://github.com/samtools/bcftools) was used to fix erroneous non-polymorphic sites in the reference sequence. 10x read cloud polishing was iteratively applied in two rounds.

Finally, we renamed the polished super-scaffolds to chromosomes according to maximum homology with the *Oreochromis niloticus*^93^ and *Amphilophus citrinellus*^3^ cichlid assemblies. Effectively, we aligned our assembly using *LASTZ* and renamed our superscaffolds^94^, ignoring smaller translocations, according to whichever previously assembled chromosome contained the majority of the *H. cyanogutatus* chromosome’s sequence.

### Reference genome annotation

Annotation of *H. cyanoguttatus* repeat sequences were predicted using RepeatMasker (v. 4.0.7) with default TE DFAM database and a de novo repeat library was then constructed using RepeatModeler (v.1.0.10)^95^. This latter was run with default parameters using the RECON (v.1.0.8) and RepeatScout (v.1.0.5) repeat finding programs, and the rmblast (v.2.6.0) search engine. This strategy allowed us to identify 35.53% of the *H. cyanoguttatus* genome as repetitive that was subsequently soft-masked for downstream analyses.

To annotate protein coding loci, total RNA was isolated from brain, eyes, fins, gonads, jaw, liver, trunk muscles and spleen (1 adult individual), and from pools of 1-day and 10-day post hatch larvae using a RNeasy Mini Kit (Qiagen). RNA quality and quantity were assessed using a Bioanalyzer 2100 (Agilent Technologies) and a Qubit v2.0 fluorometer (Life Technologies), respectively. High-quality RNA samples (RIN value > 8) were used to construct both RNA-Seq short-read and long-read libraries. For the short-reads, individual libraries for each tissue and the larval stages were constructed using a SENSE mRNA-Seq Library Prep Kit v2 (Lexogen) following Lexogen’s documentation and paired-end (2×151 bp) sequenced in an Illumina lane of a HiSeq X-Ten platform at the Beijing Institute of Technology (BGI). For the long-reads, RNA of each sample was equimolarly pooled before constructing a single cDNA Oxford Nanopore library using the Direct cDNA Sequencing Kit following manufacturer’s conditions. The single library was then sequenced in a MinION device (FLO-MIN106 9.4.1 flowcell).

Subsequently, short-reads that were filtered and corrected using Trimmomatic (v.0.39)^96^ and RCorrector (v.1.0.2)^97^ were used for de novo and reference-guided assembly. For de novo assembly, we followed the Oyster River Protocol (v.2.3.3)^98^. Briefly, clean reads were assembled using Trinity (v.2.8.5) (k-mer=25), SPAdes (v.3.13.3) (k-mer=55 and 75) and Trans-Abyss (v.2.0.1) (k-mer=32), respectively. The resulting assemblies were then merged by the OrthoFuser module. For reference-guided assembly, all clean reads were aligned to the *H. cyanoguttatus* genome, each sample independently, using the splice-aware aligner HISAT2 (v.2.1.0)^99^ with the maximum intron length set to 1 Mbp. The resulting mapping files, converted to BAM format and sorted by coordinates with Samtools (v.1.9)^100^, were processed and assembled using Trinity in the genome-guided (GG) mode. Completeness of the de novo and reference-guided assembled transcriptomes were assessed with BUSCO (v.3) using the Core Vertebrate Genes (CVG) and the Vertebrata genes (vertebrata_odb9 database) in the gVolante webserver^101^. Long-read raw output of the Oxford Nanopore MinION device was processed with Pychopper (v.2)^102^ in default mode in order to identify, orient and trim full-length Nanopore cDNA sequences.

Models for protein-coding genes were then generated following the workflow implemented in the Funannotate (v.1.8.1)^103^ automatic genome annotation pipeline while customizing several steps to improve prediction of genes in our species and optimize computational resources. First, the Funannotate *train* module, built around the PASA annotation package^104^, was run on the soft-masked version of the *H. cyanoguttatus* genome (see “Genome Masking” paragraph) taking as input: (1) the trimmed and error-corrected short RNA-Seq reads, (2) the set of transcripts generated by combining the de novo and genome-guided assemblies, further processed using the seqClean tool (https://sourceforge.net/projects/seqclean) in order to remove poly-A tails and other contaminant sequences, and (3) the full-transcript Nanopore cDNA reads. This module produced a set of 35,539 models, from which 3713 high-confidence genes were selected (see Funannotate manual for details about the filtering procedure) and used to train the gene prediction programs implemented in the following steps guided by the Funannotate *predict* module. During this core phase, gene prediction was independently carried out in training mode by Augustus (v.3.3) (16,916 genes predicted, of which14,588 were of high-quality, HiQ, with >90% exon evidence), SNAP (76,814 genes) and GlimmerHMM (92,878 genes), and in self-training mode by GeneMarkES (v.4.72) (96,877 genes). Additionally, homology-based gene evidence was determined by aligning manually curated proteins from UniProtKB/SWISSPROT database^105^ and protein sequences (ENSEMBL, release 91) of seven species to the reference genome using Exonerate (v.2.2)^106^: Nile tilapia (*Oreochromis niloticus*), three-spined stickleback (*Gasterosteus aculeatus*), zebrafish (*Danio rerio*), spotted gar (*Lepisosteus oculatus*), chicken (*Gallus gallus*), mouse (*Mus musculus*), and human (*Homo sapiens*). Additional transcript-based evidence was produced using Stringtie (v.1.3.6)^107^ by reconstructing genes from individual alignment files generated by mimimap2 for the transcripts and Hisat2 for RNA-Seq short reads. These eight independent lines of evidence were then combined with EvidenceModeler (v.1.1.1)^108^ using different weights that reflect their confidence (Augustus: 1; HiQ: 2; SNAP: 1; GlimmerHMM: 1; GeneMark: 1; PASA: 6, Exonerate/proteins: 2; Stringtie/transcripts: 1).

EvidenceModeler rendered a total of 27,982 gene models that were processed by the PASA pipeline to add untranslated region (UTR) annotations and models for alternatively spliced isoforms. Two rounds of the PASA update step were carried out. The updated gene set was then filtered by removing low-confidence isoforms. For instance, an alternative transcript for a given gene was removed when its expression was lower than 10% of the expression of the transcript with the highest expression for the same locus/gene. Expression of each transcript was measured using the whole RNA-Seq dataset and the pseudoalignment algorithm implemented in Kallisto (v.0.46.1)^109^. Low-quality gene models were removed by applying three further quality-filtering steps in an iterative fashion: (1) single-exon genes were retained only when no similarity with exons of multi-exonic genes was found (similarity was identified with the glsearch36 module implemented in the FASTA (v.36.3.8 g package95 with e-value cutoffs of 1e^-10^ and identity cutoffs of 80); (2) genes intersecting repeat elements were removed when >50% (single-exonic genes) and >90% (multi-exonic genes) were covered by repeats; (3) genes with internal stop codon(s) were removed. The completeness of the predicted protein-coding gene set was assessed with BUSCO using the Core Vertebrate Genes (CVG) and the Vertebrata genes (vertebrata_odb9 database) in the gVolante webserver. To functionally annotate the predicted gene models, we used BLASTp (-evalue 1e-5; -outfmt 6; -show_gis; -word_size 3; -num_alignments 20; -max_hsps 20) to align the translated protein sequences of the predicted genes to the UniProtKB/SWISSPROT database.

### Population genomics

We generated whole genome re-sequence data at a target of ~20x per individual coverage for 70 *H. minckleyi* and 20 *H. cyanoguttatus* (Supplementary Table 1). The two LPJ morphologies of interest were both represented relatively evenly among samples obtained from each locality (see above). We sequenced a total of 27 molariform and 29 papilliform *H. minckleyi* that were phenotyped. Fourteen additional cichlids from Cuatro Ciénegas and 20 *H. cyanoguttatus* from outside of the Cuatro Ciénegas were also genome resequenced. For this sequencing, high-molecular-weight DNA was extracted from fin or muscle tissue using a Dneasy Blood & Tissue Kit (Qiagen) while including an RNase A treatment step. DNA integrity was verified on agarose gels and concentrations were determined on a Qubit4 fluorometer. Genomic libraries were prepared with Illumina TruSeq DNA Nano kits targeting insert sizes of 350-bp and then paired-end sequenced (2 × 150 bp) on Illumina HiSeq platforms at the Beijing Genome Institute. Four individuals were pooled per lane with the aim of generating the approximate genome coverage. Raw reads were quality-controlled using Trimmomatic v0.39 and then mapped to the *H. cyanoguttatus* reference genome (fHerCya_m5) with bwa-mem (v.0.7.17)^100^. PCR duplicates were annotated with Picard Tools (v.2.7.1) (*MarkDuplicates*). Averaged across all samples, reads mapped at a coverage of ~17.4x per site.

Variant discovery and genotype calling were performed while considering all samples together using Freebayes (v.1.3.1)^91^ with standard quality filters (a minimum mapping quality 30 and a minimum base quality of 20)^110^. To speed up variant calling, we ran Freebayes in parallel over separate 5 Mb regions spanning the genome and then concatenated the resulting VCFs representing the 24 nuclear chromosomes (total length = 873,318,499 bp) into a single file with bcftools (v.1.3.1)^92^ concat. Hard quality filters were applied using the vcffilter script from the vcflib package (https://github.com/vcflib/vcflib) (command: -s -f “QUAL > 1 & QUAL / AO > 10 & SAF > 0 & SAR > 0 & RPR > 1 & RPL > 1”). The vt tools *normalize* and *uniq*^109^ were then applied to normalize variants and remove duplicates. Further variant filtering was conducted to set individual genotypes with sequencing depth less than 10x or greater than 50x (approximately twice the raw mean depth per sample) and genotype quality less than 30 as missing. We also included only SNPs with minor alleles present more than once, excluded any sites at which no alternate alleles remained after filtering or where only alternate alleles were called, and removed possible false positive singletons by excluding sites with a minor allele frequency of less than or equal to 5%. Lastly, we excluded sites with more than 20% missing data and/or that were not biallelic SNPs. Linkage disequilibrium (LD) was calculated with bcftools (v.1.3.1)^92^ and the +prune plugin to calculate *r*^2^ values across sites in windows of 100 kb in order to exclude SNPs in high LD (*r*^2^ > 0.5) for population genomic inferences.

We assessed gene flow between *H. minckleyi* and *H. cyanoguttatus* and the genetic structure among different populations (i.e., pools) of *H. minckleyi* endemic to the Cuatro Ciénegas Basin using several strategies. First, a genomic principal components analysis (PCA) was carried out on the LD-pruned SNPs with PLINK (v.1.90)^111^ to assess phylogenetic clustering. Second, to visualize the underlying phylogenetic structure of these SNPs, a distance matrix was first produced from an imputed version of the LD-pruned dataset (phased with Beagle (v.5.1))^112^ using the python scripts parseVCF.py and distMat.py (available at: https://github.com/simonhmartin/genomics_general) and then a phylogenetic neighbor-network was reconstructed from the resulting matrix in SplitsTree (v.4.17.0)^113^. Third, the genetic admixture of each sample in the LD-pruned dataset was determined with ADMIXTURE (v.1.3.0)^114^ by examining K = 1–10 groups. We compared the cross-validation (CV) errors of the ten tests to identify the optimal grouping assignment (that with the lowest CV error). Results from these analyses were plotted in R. Finally, the mean genome-wide Weir-Cockerham *Fst* values were estimated between the different *H. minckleyi* populations and *H. cyanoguttatus* with vcftools (v.0.1.15)^92^ to estimate the amount of interbreeding. The linear geographic distances were calculated from geographic coordinates (Supplementary Table 5) between *H. minckleyi* sampled populations^115^.

### Genotype to tooth phenotype mapping

To isolate the genomic basis of *H. minckleyi* tooth size, a parental papilliform *Herichthys minckleyi* (collected from Cuatro Ciénegas) and several molariform *H. cyanoguttatus* (purchased commercially) were maintained together in a single aquarium. Animal husbandry was approved by the German authorities (permit number G19-051, Regierungspräsidium Freiburg, Abteilung 3, Referat 34, Veterinärwesen & Lebensmittelüberwachung, Germany). We have complied with all relevant ethical regulations for animal use. The single male individual (*H. minckleyi*) was placed along with several *H. cyanoguttatus* females in a 40 L aquarium. The male successfully fertilized the eggs of one of the *H. cyanoguttatus* females after a duration of several weeks. The parentals offspring (F1 generation individuals) were removed after hatching and moved to larger 80 Liter aquarium tanks to be allowed to grow to sexual maturity. The F1 generation were maintained together and allowed to inter-cross (F1 X F1) to produce several broods to form our second hybrid generation (F2) for subsequent QTL mapping.

Fin clips of the two parental individuals and all F2 offspring were taken from their caudal fins ( ~ 25 mm^2^ of tissue). The F2 hybrids were all sampled when they achieved at least 75 mm in total length when the fish were ~1 year of age. Each fin clip was preserved in 100% ETOH with a unique tag number that was also used to tag the individual F2 specimens. Following fin-clipping and tagging, fish were sacrificed with an overdose of tricaine methane sulfonate (MS-222). The fin clips were then used in genomic library preparation following individual DNA extraction.

To assess genotypes, genomic DNA was isolated from fin tissues of the 198 F2, four F1, and both parental individuals using the Zymo extraction kit (Zymo Research). DNA integrity was evaluated both with agarose gel electrophoresis and a QUBIT v2.0 fluorometer (Life Technologies). Then, to generate RADseq libraries, we followed a ddRAD methodology^116^ with some updated modification^117^. Sequence fragment size selection was performed using Pippin Prep (Sage Science) with a range of 325–400 bp. Individually barcoded samples were then genotyped in pooled Illumina libraries. All genomic libraries were single-end sequenced (101 bp length) on an Illumina HiSeq 2000 platform at the genomic facility of the University of Tufts (TUCF Genomics).

For linkage map construction, we used ddRAD data for the whole sample set (F2s and parents of the cross) and reads from whole-genome-resequencing of the parents (Supplementary data 1). For ddRAD reads, barcode demultiplexing and filtering out low-quality reads was performed with the *process_radtags* module implemented in Stacks (v.1.35)^118^. For WGS, raw reads of each parental individual were quality-controlled using Trimmomatic (v.039)^96^. RAD and WGS reads were then aligned to the *H. cyanoguttatus* reference genome using bwa-mem (v.0.7.15)^100^ with default settings for either single- and paired-end mapping modes. MarkDuplicates module of PicardTools (v.1.141) was used to remove duplicates in WGS aligned reads. Using the individuals’ alignments as input, genotypes were called using Freebayes (v.1.3.1)^91^. For further analysis, only those markers for which parentals were homozygous for different alleles, and for which F2 genotype frequencies followed the expected segregation ratio (χ2 test; *P* = 0.05 threshold) were retained. We only included individuals with at least 90% called genotypes and loci present in at least 90% of the individuals. The linkage map was constructed with a regression-based algorithm implementing the Kosambi mapping function in JoinMap (v.5)^119^. The final linkage map had a total inferred size of 1507.92 cM and was blanketed with 3087 markers distributed across 24 linkage groups.

To determine pharyngeal jaw phenotypes, we dissected the fifth ceratobranchial from the fish. These bony elements were then cleaned of all muscle and fascia and allowed to dry. Then, a size-standardized digital image of the dorsal surface of the jaws was taken and imported into the program ImageJ (v.1.51)^83^. Because it allowed the strongest inference of homology among individuals, we measured the posterior-most tooth to the right of the pharyngeal jaw midline to determine pharyngeal tooth area. To size standardize the values, tooth area measurements were square root transformed and divided by SL and subsequently used in the QTL analyses. Candidate QTL loci were identified using the *scanone* function with Haley-Knott regression and added to a multiple-QTL model using the *fitqtl* function in *r/qtl* (v.1.50)^120^. Models were evaluated using multiple regression *F*-tests (*P* > 0.05) and model selection proceeded by dropping non-significant QTL. Finally, the obtained model was refined using the *refineqtl* function, and confidence intervals (CI) for each QTL were inferred using the *bayesint* function. Subsequently, the physical location in the genome of markers bracketing the CI were determined.

Genome-wide association mapping was then performed to clarify the genomic basis of the *H. minckleyi* dental polymorphism. To perform the genomic associations, we first used PLINK to generate the genotype input files account for population location and phenotype^111^. Using our VCF from the gene flow analyses above, we excluded all individuals that did not have a pharyngeal phenotype, markers with more than 20% missing data, and markers with a minor allele frequency less than 0.05. Then, we used gemma (v.3.2.0)^121^ to estimate a kinship matrix to account for population structure and implement a univariate linear mixed model to perform the association between genotypes and tooth phenotype. The lowest genome-wide empirical *P*-values based on the Wald statistic were then obtained.

### Sliding window D-statistics

Because the largest QTL peak and the genome-wide association mapped to the same physical region, we examined the extent of introgression from *H. cyanoguttatus* into each of the *H. minckleyi* pharyngeal morphotypes on chromosome 11. To estimate this, we calculated D-statistics (also referred to as ABBA–BABA statistics) across sliding windows of variable SNPs using the Dinvestigate tool in the program Dsuite v0.4 r38^122^. This analysis was used to determines if there was an excess of either ABBA or BABA evolutionary patterns in the SNPs present in either the papilliform or molariform *H. minckleyi* (Supplementary Fig. 3). The whole genome resequenced data from all morphologically phenotyped molariforms, all phenotyped papilliforms, and all *H. cyanoguttatus* were used in this analysis. Then, we used a sequence of the close outgroup^40^ to *H. minckleyi* and *H. cyanoguttatus*, *Herichthys pame*, to polarize the Dsuite analyses. If either group of the papilliform or molariform *H. minckleyi* shared more SNP alleles than expected with *H. cyanoguttatus* in a particular region, this would be consistent with introgression from *H. cyanoguttatus*. We examined a range of step sizes (5 to 20) and sliding genomic windows (20 to 100) across the entire chromosome for context, but particularly focused on the region identified on Chromosome 11 where the QTL and genome-wide association overlapped.

### Genomic basis of the polymorphism

Using the annotated *H. cyanoguttatus* genome as a reference, the nearby protein-coding loci were identified for the most divergent SNPs between the morphotypes. The genotype frequencies in the molariform and papilliform *H. minckleyi* polarized by the genotypes present in *H. cyanoguttatus* were also examined in a physical window 100 SNPs upstream and 100 SNPs downstream of the most highly associated SNP (Supplementary data 2). Summary statistics for the top five SNPs associated with tooth area were especially focused upon and genotypes for all *H. cyanoguttatus* individuals from outside of Cuatro Ciénegas as well as those for the molariform and papilliform *H. minckleyi* were reported (Table 2). Because of the deviations in genotypes observed at these highly associated loci, chi-square tests for deviations from Hardy-Weinberg equilibrium (HWE) and Cramer’s V measures of effect size were examined treating the papilliform and molariform *H. minckleyi* as their own distinct population in this single genomic region.

### Reporting summary

Further information on research design is available in the Nature Portfolio Reporting Summary linked to this article.

## Supplementary information

Supplementary Information

Description of Additional Supplementary File

Supplemental Data 1-2

Reporting Summary

## Data Availability

The *Herichthys cyanogutattus* genome assembly, the genomic data used to build and scaffold the contigs (PacBio HiFi long reads, 10x chromium synthetic long reads, Hi-C short reads, and Bionano optical map), and the transcriptomic data used to annotate the protein-coding genes in the genome have been deposited into the NCBI database under BioProject PRJNA1163081. The ddRAD data of the two parents and the 193 F2 individuals used to produce the linkage map and perform QTL mapping of the tooth area have been deposited into the NCBI database under BioProject PRJNA1165996. The WGS data of the 70 individuals, including the two parents of the QTL cross, used to analyze population structure, gene flow, and carry out GWAS, have been deposited into the NCBI database under BioProject PRJNA1167894. The *Herichthys cyanogutattus* protein-coding gene annotation^123^ has been deposited into FigShare.
