# Supplementary material for: Divergence at the IRX gene cluster underlies extreme trophic polymorphism in a cichlid fish (Herichthys minckleyi)
Source: Commun Biol. 2026 Feb 21;9:508. doi: 10.1038/s42003-026-09689-6 (PMC13066147; doi:10.1038/s42003-026-09689-6)
Supplement: Supplementary file 1 — Supplementary Information [file 42003_2026_9689_MOESM1_ESM.pdf]

Supporting information for

Divergence at the *IRX* gene cluster underlies extreme trophic polymorphism in a cichlid fish (*Herichthys minckleyi*)

C. Darrin Hulsey*[1,2], Paolo Franchini[2,3], Paul Masonick[2], Andreas Kautt[4], Gonzalo Machado-Schiaffino[2,5], Martin Pippel[6], Francisco García de León[7], Eugene Myers[6], and Axel Meyer*[2,8]

[1]School of Biology and Environmental Science, University College Dublin, Dublin, Ireland

[2]Department of Biology, University of Konstanz, Konstanz, Germany

[3]Department of Ecology and Biology (DEB), Tuscia University, Viterbo, Italy

[4]Department of Biology, Washington University, St. Louis, MO, USA

[5]Department of Functional Biology, Area of Genetics, University of Oviedo, Oviedo, Spain

[6]Systems Biology Center, Max Planck Institute of Molecular Cell Biology and Genetics, Dresden. Germany

[7]Laboratorio de Genética para la Conservación, Centro de Investigaciones Biológicas del Noroeste, La Paz, BCS, Mexico

[8]Museum of Comparative Zoology, Harvard University, Cambridge, MA, USA

*Corresponding authors
E-mails: darrin.hulsey1@ucd.ie; axel.meyer@uni-konstanz.de

This file includes:

Figures S1 to S3

Tables S1 to S6

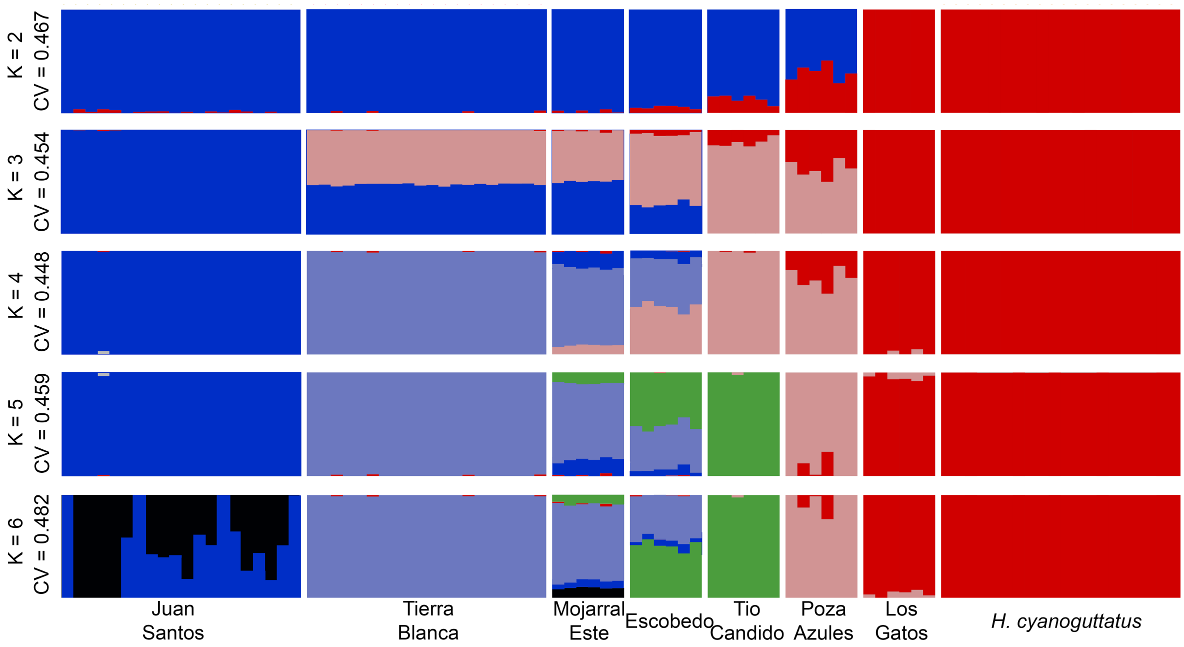

Supplemental Figure 1. Admixture Plots. Admixture analyses were run with K = 1 to K = 10 population subdivisions. A good value of K exhibits a low cross-validation (CV) error compared to other K values and once the lowest value (best supported) is reached, K tends to increase with further subdivision. The CV value for a single panmictic population, K = 1, began at 0.571 and CV values as K increased from 2 to 6 are shown to the left of their respective admixture plots. Notably, K = 2 primarily differentiated cichlids with a *H. minckleyi* genome from individuals with a more *H. cyanoguttatus* genomic background. A value of K = 4 had the lowest CV and is reproduced in Figure 2 in the main text.

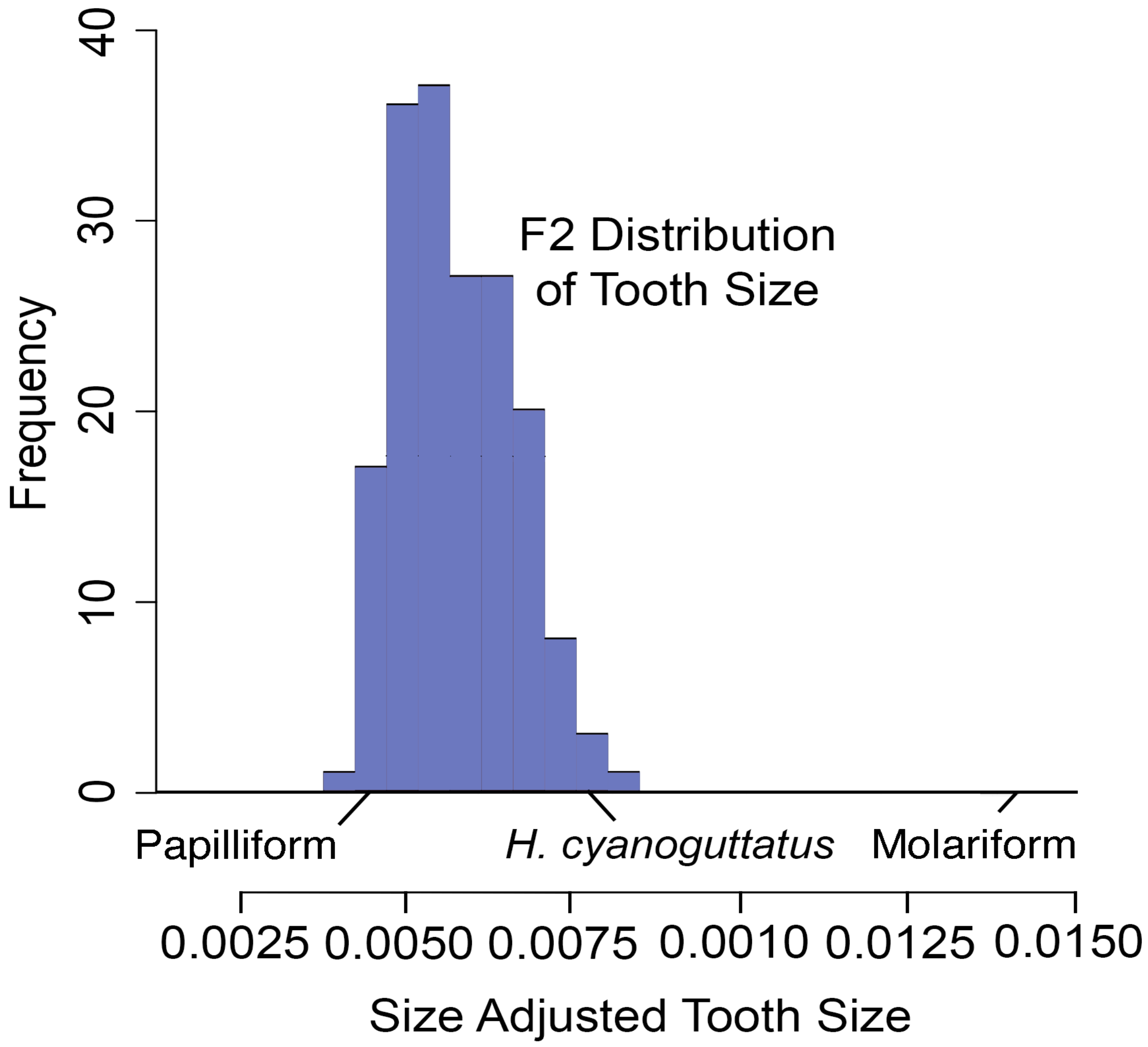

Supplemental Figure 2. F2 Tooth Size Phenotypic Distribution. The distribution of pharyngeal tooth sizes in our F2 cross was normally distributed. Mean values for tooth size for papilliform *H. minckleyi*, *H. cyanoguttatus*, and molariform H. minckleyi are depicted. There was little evidence of transgressive phenotypes in the hybrid cross, as the F2 pharyngeal tooth sizes generally ranged between the average value for a papilliform, the father, and the average value for *H. cyanoguttatus,* the mother. The molariform *H. minckleyi* tooth sizes were much larger on average than teeth in the F2.

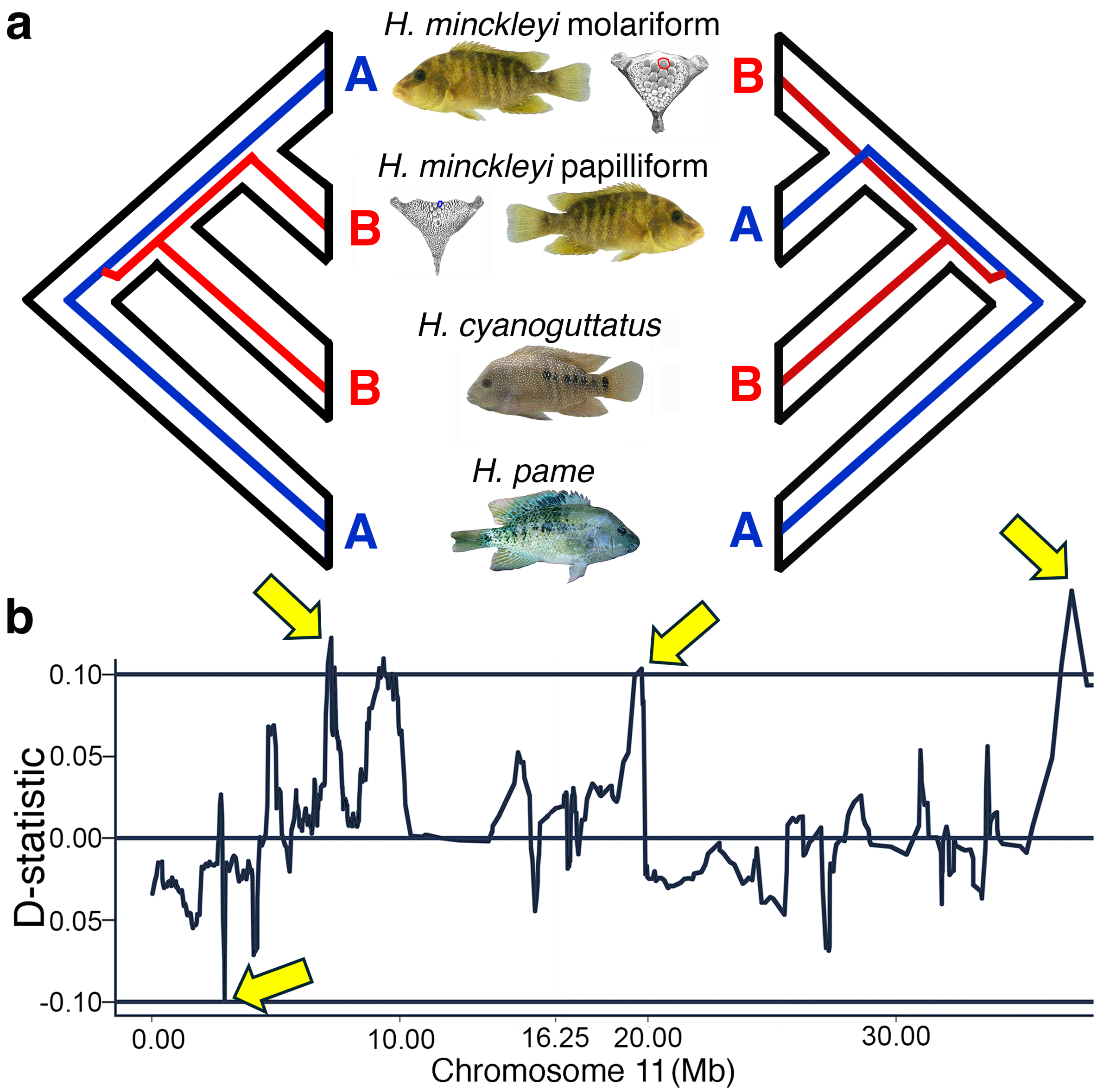

Supplemental Figure 3. Sliding Window D-Statistics. **a**, We calculated D-statistics, also referred to as ABBA–BABA statistics, across sliding windows using the Dinvestigate tool in the program Dsuite v0.4 r38 (Malinsky et al. 2021). This analysis was used to determines if there was an excess of either ABBA or BABA evolutionary patterns in the SNPs present in either the papilliform or molariform *H. minckleyi*. If one of these patterns was more common than expected, this would indicate an excess of SNP allele sharing from *H. cyanoguttatus* as polarized by the outgroup *H. pame*. **b**, We examined a range of step sizes and sliding genomic windows and focused on the region identified on Chromosome 11 where the QTL and genome-wide association overlapped. The yellow arrows point to elevated D-statistics in either the molariforms or papilliforms that would be expected if introgression *from H. cyanoguttatus* characterized the region of high genomic association associated with the *H. minckleyi* morphotypes. This was not the pattern recovered.

Supplemental Table 1. *Herichthys* samples*.* Individuals sampled for genomic and phenotypic analyses are listed. Individual ID associated with whole genome re-sequecing files, their collection location, pharyngeal morphotype (M = molariform and P = papilliform), phenotyped values of size-adjusted tooth area for *H. minckleyi* individuals, and Genbank short read archive accession numbers are detailed.

ID	Collection Location	Morph	Tooth Size	Accession
3434	Juan Santos, Cuatro Ciénegas, Coahuila	M	0.01410	SAMN54469540
3435	Juan Santos, Cuatro Ciénegas, Coahuila	M	0.01311	SAMN54469541
3442	Juan Santos, Cuatro Ciénegas, Coahuila	M	0.01149	SAMN54469543
3643	Juan Santos, Cuatro Ciénegas, Coahuila	M	0.01343	SAMN54469545
3644	Juan Santos, Cuatro Ciénegas, Coahuila	M	0.01247	SAMN54469546
3645	Juan Santos, Cuatro Ciénegas, Coahuila	M	0.01424	SAMN54469547
3646	Juan Santos, Cuatro Ciénegas, Coahuila	M	0.01555	SAMN54469548
3647	Juan Santos, Cuatro Ciénegas, Coahuila	M	0.00874	SAMN54469549
3655	Juan Santos, Cuatro Ciénegas, Coahuila	M	0.01145	SAMN54469551
3660	Juan Santos, Cuatro Ciénegas, Coahuila	M	0.01303	SAMN54469553
3154	Tierra Blanca, Cuatro Ciénegas, Coahuila	M	0.01157	SAMN54469556
3168	Tierra Blanca, Cuatro Ciénegas, Coahuila	M	0.01818	SAMN54469561
3181	Tierra Blanca, Cuatro Ciénegas, Coahuila	M	0.01108	SAMN54469567
3266	Tierra Blanca, Cuatro Ciénegas, Coahuila	M	0.01439	SAMN54469568
3272	Tierra Blanca, Cuatro Ciénegas, Coahuila	M	0.01899	SAMN54469569
3273	Tierra Blanca, Cuatro Ciénegas, Coahuila	M	0.01887	SAMN54469570
3274	Tierra Blanca, Cuatro Ciénegas, Coahuila	M	0.01425	SAMN54469571
3275	Tierra Blanca, Cuatro Ciénegas, Coahuila	M	0.01738	SAMN54469572
3278	Tierra Blanca, Cuatro Ciénegas, Coahuila	M	0.01953	SAMN54469574
3280	Tierra Blanca, Cuatro Ciénegas, Coahuila	M	0.01578	SAMN54469575
3363	Tio Candicdo, Cuatro Ciénegas, Coahuila	M	0.01253	SAMN44016764
3383	Tio Candicdo, Cuatro Ciénegas, Coahuila	M	0.00784	SAMN44016776
3535	Escobeda, Cuatro Ciénegas, Coahuila	M	0.01972	SAMN44016790
3543	Escobeda, Cuatro Ciénegas, Coahuila	M	0.00855	SAMN44016793
3570	Escobeda, Cuatro Ciénegas, Coahuila	M	0.01146	SAMN44016796
3670	Mojarral Este, Cuatro Ciénegas, Coahuila	M	0.01499	SAMN44016806
3677	Mojarral Este, Cuatro Ciénegas, Coahuila	M	0.01463	SAMN44016812
3426	Juan Santos, Cuatro Ciénegas, Coahuila	P	0.00383	SAMN54469536
3428	Juan Santos, Cuatro Ciénegas, Coahuila	P	0.00389	SAMN54469537
3429	Juan Santos, Cuatro Ciénegas, Coahuila	P	0.00473	SAMN54469538
3431	Juan Santos, Cuatro Ciénegas, Coahuila	P	0.00376	SAMN54469539
3436	Juan Santos, Cuatro Ciénegas, Coahuila	P	0.00408	SAMN54469542
3642	Juan Santos, Cuatro Ciénegas, Coahuila	P	0.00474	SAMN54469544
3652	Juan Santos, Cuatro Ciénegas, Coahuila	P	0.00579	SAMN54469550
3658	Juan Santos, Cuatro Ciénegas, Coahuila	P	0.00443	SAMN54469552
3661	Juan Santos, Cuatro Ciénegas, Coahuila	P	0.00500	SAMN54469554
3683	Juan Santos, Cuatro Ciénegas, Coahuila	P	0.00411	SAMN54469555
3162	Tierra Blanca, Cuatro Ciénegas, Coahuila	P	0.00523	SAMN54469557
3164	Tierra Blanca, Cuatro Ciénegas, Coahuila	P	0.00368	SAMN54469558
3165	Tierra Blanca, Cuatro Ciénegas, Coahuila	P	0.00481	SAMN54469559
3166	Tierra Blanca, Cuatro Ciénegas, Coahuila	P	0.00430	SAMN54469560
3171	Tierra Blanca, Cuatro Ciénegas, Coahuila	P	0.00360	SAMN54469562

3172	Tierra Blanca, Cuatro Ciénegas, Coahuila	P	0.00393	SAMN54469563
3175	Tierra Blanca, Cuatro Ciénegas, Coahuila	P	0.00404	SAMN54469564
3176	Tierra Blanca, Cuatro Ciénegas, Coahuila	P	0.00550	SAMN54469565
3178	Tierra Blanca, Cuatro Ciénegas, Coahuila	P	0.00387	SAMN54469566
3276	Tierra Blanca, Cuatro Ciénegas, Coahuila	P	0.00458	SAMN54469573
3366	Tio Candicdo, Cuatro Ciénegas, Coahuila	P	0.00412	SAMN44016767
3373	Tio Candicdo, Cuatro Ciénegas, Coahuila	P	0.00453	SAMN44016770
3376	Tio Candicdo, Cuatro Ciénegas, Coahuila	P	0.00433	SAMN44016773
3518	Escobeda, Cuatro Ciénegas, Coahuila	P	0.00335	SAMN44016781
3521	Escobeda, Cuatro Ciénegas, Coahuila	P	0.00422	SAMN44016784
3525	Escobeda, Cuatro Ciénegas, Coahuila	P	0.00344	SAMN44016787
3664	Mojarral Este, Cuatro Ciénegas, Coahuila	P	0.00592	SAMN44016800
3667	Mojarral Este, Cuatro Ciénegas, Coahuila	P	0.00560	SAMN44016803
3674	Mojarral Este, Cuatro Ciénegas, Coahuila	P	0.00494	SAMN44016809
2447	Rio San Rafael, Tamaulipas	NA	NA	SAMN44016706
2448	Rio San Rafael, Tamaulipas	NA	NA	SAMN44016707
2474	Rio Purificacion, Tamaulipas	NA	NA	SAMN44016708
2475	Rio Purificacion, Tamaulipas	NA	NA	SAMN44016709
2477	Rio Purificacion, Tamaulipas	NA	NA	SAMN44016710
2478	Rio Purificacion,Tamaulipas	NA	NA	SAMN44016711
2479	Rio Purificacion, Tamaulipas	NA	NA	SAMN44016712
2557	Devils River, Texas	NA	NA	SAMN44016713
2559	Devils River, Texas	NA	NA	SAMN44016714
3106	Rio Salado de los Nadadores, Coahuila	NA	NA	SAMN44016715
3107	Rio Salado de los Nadadores, Coahuila	NA	NA	SAMN44016716
3112	Rio Salado de los Nadadores, Coahuila	NA	NA	SAMN44016717
3113	Rio Salado de los Nadadores, Coahuila	NA	NA	SAMN44016718
3114	Rio Salado de los Nadadores, Coahuila	NA	NA	SAMN44016719
3115	Rio Salado de los Nadadores, Coahuila	NA	NA	SAMN44016720
3116	Rio Salado de los Nadadores, Coahuila	NA	NA	SAMN44016721
3126	Rio Salado de los Nadadores, Coahuila	NA	NA	SAMN44016722
3135	Rio Salado de los Nadadores, Coahuila	NA	NA	SAMN44016723
3136	Rio Salado de los Nadadores, Coahuila	NA	NA	SAMN44016724
2956	Mojarral Este, Cuatro Ciénegas, Coahuila	NA	NA	SAMN44016750
2343	Los Gatos, Cuatro Ciénegas, Coahuila	NA	NA	SAMN44016736
2352	Los Gatos, Cuatro Ciénegas, Coahuila	NA	NA	SAMN44016739
2354	Los Gatos, Cuatro Ciénegas, Coahuila	NA	NA	SAMN44016741
2356	Los Gatos, Cuatro Ciénegas, Coahuila	NA	NA	SAMN44016743
2358	Los Gatos, Cuatro Ciénegas, Coahuila	NA	NA	SAMN44016745
2360	Los Gatos, Cuatro Ciénegas, Coahuila	NA	NA	SAMN44016747
3094	Poza Azules, Cuatro Ciénegas, Coahuila	NA	NA	SAMN44016753
3098	Poza Azules, Cuatro Ciénegas, Coahuila	NA	NA	SAMN44016754
3099	Poza Azules, Cuatro Ciénegas, Coahuila	NA	NA	SAMN44016755
3100	Poza Azules, Cuatro Ciénegas, Coahuila	NA	NA	SAMN44016756
3102	Poza Azules, Cuatro Ciénegas, Coahuila	NA	NA	SAMN44016757
3104	Poza Azules, Cuatro Ciénegas, Coahuila	NA	NA	SAMN44016758
3359	Tio Candido, Cuatro Ciénegas, Coahuila	NA	NA	SAMN44016761

Supplemental Table 2. **Central American cichlids**. Species for which the mean of size-standardized pharyngeal tooth size was measured and the among species variation subsequently compared to tooth size variation within *H. minckleyi*.

Species	n	Tooth Size
Amatitlania nigrofasciata	1	0.00538
Amatitlania septemfasciata	1	0.00647
Amphilophus citrinellus	1	0.00998
Amphilophus trimacultatus	3	0.00862
Archocentrus centrarchus	6	0.00592
Astatheros macracanthus	2	0.00821
Chuco intermedium	1	0.00778
Cincelichthys pearsei	1	0.00491
Criboheros longimanus	1	0.00548
Cribroheros robertsoni	3	0.00547
Darienheros calobrensis	1	0.00631
Herichthys bartoni	3	0.00559
Herichthys deppii	1	0.00650
Herichthys labridens	4	0.01371
Herichthys pantostictus	2	0.00818
Herichthys tamasopoensis	2	0.00481
Hypsophrys nigaraguensis	1	0.00464
Mayaheros urophthalmus	5	0.00753
Parachromis loisellei	1	0.00547
Pareneetroplus bulleri	3	0.00463
Petenia splendida	3	0.00429
Rocio octofasciata	5	0.00689
Theraps irregularis	1	0.00647
Thorichthys aureus	1	0.00908
Thorichthys callolepis	3	0.00572
Thorichthys ellioti	3	0.00474
Thorichthys helleri	3	0.00541
Thorichthys pasionis	3	0.00745
Trichromis salvini	2	0.00406
Veija maculicauda	8	0.00796
Vieja fenestrata	1	0.00632
Vieja guttulata	2	0.00702
Vieja synspila	1	0.00761

Supplemental Table 3. *Herichthys cyanoguttatus* Genome Statistics. Statistics on the genome assembly, BUSCO analysis of the genome, protein coding gene annotation, and BUSCO analysis on predicted protein coding genes.

**Genome Assembly**			
Chromosomes	24		
Number of scaffolds	94		
Number of contigs	499		
Total length	889514973		
Percent gaps	0.01%		
Scaffold N50	37 Mbp		
Contigs N50	5 Mbp		
**BUSCO analysis on genome:**			
BUSCO version			6.0.0
Lineage dataset			actinopterygii_odb12
C, complete genes. S, Single copy genes. D, duplicated genes, F, fragmented genes. M, missing genes			C:99.4%[S:99.0%,D:0.4%],F:0.1%,M:0.5%
Complete BUSCOs (C)			7165
Complete and single-copy BUSCOs (S)			7133
Complete and duplicated BUSCOs (D)			32
Fragmented BUSCOs (F)			5
Missing BUSCOs (M)			37
Total BUSCO groups searched			7207
**Protein Coding Gene Annotation:**			
**Type**	**Number**	**Size total (kb)**	**Size mean (bp)**
cds	271572	46369.19	170.74
exon	278166	55350.48	198.98
gene	25769	433025.91	16804.14
mrna	28343	511522.97	18047.59
Total	603850	1046268.56	1732.66
**BUSCO analysis on predicted protein coding genes:**			
BUSCO version			6.0.0
Lineage dataset			actinopterygii_odb12
C, complete genes. S, Single copy genes. D, duplicated genes, F, fragmented genes. M, missing genes.			C:92.4%[S:86.5%,D:5.9%],F:3.0%,M:4.6%
Complete BUSCOs (C)			6658
Complete and single-copy BUSCOs (S)			6232
Complete and duplicated BUSCOs (D)			426
Fragmented BUSCOs (F)			218
Missing BUSCOs (M)			331
Total BUSCO groups searched			7207

Supplemental Table 4. **Comparative Cichlid Genome Statistics**. Comparisons between basic genome structure of *H. cyanoguttatus* and selected other cichlid genomes available on Ensembl (https://www.ensembl.org/).

**Species**	**Location**	**Genome size (Gbp)**	**Contig NG50 (Mbp)**	**Protein coding genes**	**BUSCO**	**Repeat content**
Herichthys cyanoguttatus	Neotropics	0.89	5.0	25,769	99.4%	35.53%
Archocentrus centrachus	Neotropics	0.99	2.2	25,253	97.3%	29.37%
Amphilophus citrinellus	Neotropics	0.96	3.8	24,528	98.7%	28.81%
Oreochromis niloticus	Africa	1.01	2.9	28,189	95.4%	37.56%
Astatotilapia calliptera	Africa	0.88	4.4	25,714	97.2%	30.81%
Maylandia zebra	Africa	0.96	1.4	25,898	98.4%	28.10%

Supplemental Table 5. **Sampling locations**. Geographic coordinates used in population genomic comparisons of *Herichthys minckleyi* within Cuatro Ciénegas and for the *H. cyanoguttatus* from the Rio Salado.

Location	Latitude	Longitude
Juan Santos	26° 53.86N	102° 08.81W
Tierra Blanca	26° 52.23N	102° 08.37W
Mojarral East	26° 55.48N	102° 07.28W
Escobedo	26° 52.30N	102° 05.26W
Tio Candido	26° 52.33N	102° 04.85W
Poza Azules	26° 49.73N	102° 01.68W
Los Gatos	26° 54.89N	102° 02.54W
Rio Salado	27° 02.06N	101° 43.30W

Supplemental Table 6. Quantitative trait loci (QTL) models. Statistical results for QTL of size adjusted pharyngeal tooth area in the cross of *H. minckleyi* and *H. cyanoguttatus*. The full model and results from the drop one QTL are given as well as degrees of freedom (df), logarithm of odds (LOD), proportion of variation explained (PVE), *P*-value associated with the model, the genomic confidence interval (CI) for individual QTL, and the genomic location of physical markers bracketing each confidence interval.

	Df	***LOD***	***PVE***	***P***	***CI***	***Physical Markers (Mb)***
Full Model	8	17.75	36.65	<0.0001		
Drop one QTL						
11@58.3	2	14.44	19.38	<0.0001	11@49.5 - 11@62.0	12.64 - 26.24
10@20.8	2	7.72	10.36	<0.0001	10@20.5 - 10@50.5	10.74 - 28.44
19@25.3	2	7.61	10.21	<0.0001	19@21.5 - 19@45.0	8.89 - 18.34
5@36.0	2	5.93	7.95	0.0004	5@27.5 - 5@57.5	16.27 - 38.57
