# Supplementary material for: Divergence at the IRX gene cluster underlies extreme trophic polymorphism in a cichlid fish (Herichthys minckleyi)
Source: Commun Biol. 2026 Feb 21;9:508. doi: 10.1038/s42003-026-09689-6 (PMC13066147; doi:10.1038/s42003-026-09689-6)
Supplement: Supplementary file 2 — Description of Additional Supplementary File [file 42003_2026_9689_MOESM2_ESM.pdf]

Description of Additional Supplementary Files

File name: Supplementary Data 1

Description: Quantitative trait locus (QTL) input file. The QTL input file for the pharyngeal tooth size and F2 specimen ID are given and then genotypes arranged by chromsome number in the reference genome are given.

File name: Supplementary Data 2

Description: Association Window. Single nucleotide polymorphisms (SNPs) in a 200 SNP window surrounding the SNP showing the highest association with pharyngeal morphotype in H. minckleyi. The chromosomal position (POS), reference (REF) as well as the alternative (REF) allele scored for H. minckleyi and H. cyanoguttatus individuals are shown. Reference alleles are scored as 0 and alternative alleles as 1. When avialable, pharyngeal morphotype (Morph) of H. minckleyi are given. These genotypes were used to generate Figure 4A.
