# Supplementary material for: Divergence at the IRX gene cluster underlies extreme trophic polymorphism in a cichlid fish (Herichthys minckleyi)
Source: Commun Biol. 2026 Feb 21;9:508. doi: 10.1038/s42003-026-09689-6 (PMC13066147; doi:10.1038/s42003-026-09689-6)
Supplement: Supplementary file 4 — Reporting Summary [file 42003_2026_9689_MOESM4_ESM.pdf]

nature portfolio

Corresponding author(s): C. Darrin Hulsey

Last updated by author(s): 1/4/2026

Reporting Summary

Nature Portfolio wishes to improve the reproducibility of the work that we publish. This form provides structure for consistency and transparency in reporting. For further information on Nature Portfolio policies, see our Editorial Policies and the Editorial Policy Checklist.

Please do not complete any field with "not applicable" or n/a. Refer to the help text for what text to use if an item is not relevant to your study.
For final submission: please carefully check your responses for accuracy; you will not be able to make changes later.

Statistics

For all statistical analyses, confirm that the following items are present in the figure legend, table legend, main text, or Methods section.

n/a	Confirmed	
☐	☒	The exact sample size (*n*) for each experimental group/condition, given as a discrete number and unit of measurement
☐	☒	A statement on whether measurements were taken from distinct samples or whether the same sample was measured repeatedly
☐	☒	The statistical test(s) used AND whether they are one- or two-sided<br>*Only common tests should be described solely by name; describe more complex techniques in the Methods section.*
☒	☐	A description of all covariates tested
☐	☒	A description of any assumptions or corrections, such as tests of normality and adjustment for multiple comparisons
☐	☒	A full description of the statistical parameters including central tendency (e.g. means) or other basic estimates (e.g. regression coefficient) AND variation (e.g. standard deviation) or associated estimates of uncertainty (e.g. confidence intervals)
☐	☒	For null hypothesis testing, the test statistic (e.g. *F*, *t*, *r*) with confidence intervals, effect sizes, degrees of freedom and *P* value noted<br>*Give P values as exact values whenever suitable.*
☒	☐	For Bayesian analysis, information on the choice of priors and Markov chain Monte Carlo settings
☐	☒	For hierarchical and complex designs, identification of the appropriate level for tests and full reporting of outcomes
☒	☐	Estimates of effect sizes (e.g. Cohen's *d*, Pearson's *r*), indicating how they were calculated

Our web collection on statistics for biologists contains articles on many of the points above.

Software and code

Policy information about availability of computer code

Data collection

Pharyngeal tooth size was measured from digitial images in ImageJ v.1.51

Data analysis

Morphological Data:
ImageJ (v.1.51) / cvequality (R package v.0.2.0)

Genome Assembly and annotation:
Marvel assembler, DBdust (https://github.com/thegenemyers/DAZZ_DB), TANmask ,daligner (https://github.com/thegenemyers/DALIGNER), Bionano Solve 3.3_10252018 (https://bionanogenomics.com/support-page/bionano-solve/), SALSA2, Arima mapping pipeline (https://github.com/ArimaGenomics/mapping_pipeline), Longranger align pipeline (https://github.com/10XGenomics/longranger, v.2.2.0), freebates (v.1.2.0) bcftools consensus (v.1.9), LASTZ, RepeatModeler (v.1.0.10), rmblast (v.2.6.0), Trimmomatic (v.0.39), RCorrector (v.1.0.2), Oyster River Protocol (v.2.3.3), Trinity (v.2.8.5), SPAdes (v.3.13.3), Trans-Abyss (v.2.0.1), HISAT2 (v.2.1.0), Samtools (v.1.9), BUSCO (v.3), gVolante webserver, Pychopper (v.2), Funannotate (v.1.8.1), PASA, seqClean tool (https://sourceforge.net/projects/seqclean), UniProtKB/SWISSPROT database, Exonerate, Stringtie (v.1.3.6), EvidenceModeler (v.1.1.1), Kallisto (v.0.46.1)

Population Genomics
Trimmomatic v0.39, bwa-mem (v.0.7.17), Picard Tools (v.2.7.1), Freebayes (v.1.3.1), bcftools (v.1.3.1), PLINK (v.1.90), Beagle (v.5.1), SplitsTree (v.4.17.0) ADMIXTURE (v.1.3.0), vcftools (v.0.1.15)

QTL mapping
Stacks (v.1.35), Trimmomatic (v.039), PicardTools (v.1.141), bwa-mem (v.0.7.15), Freebayes (v.1.3.1), JoinMap (v.5), ImageJ (v.1.51), r/qtl (v.1.50)

GWA
PLINK (v.1.90), gemma (v.3.2.0)

D-statistics
Dsuite v0.4 r38

For manuscripts utilizing custom algorithms or software that are central to the research but not yet described in published literature, software must be made available to editors and reviewers. We strongly encourage code deposition in a community repository (e.g. GitHub). See the Nature Portfolio guidelines for submitting code & software for further information.

Data

Policy information about availability of data

All manuscripts must include a data availability statement. This statement should provide the following information, where applicable:

- Accession codes, unique identifiers, or web links for publicly available datasets
- A description of any restrictions on data availability
- For clinical datasets or third party data, please ensure that the statement adheres to our policy

The authors declare that all data supporting the findings of this study are available within the main text and supplementary materials. Raw data used to generate all graphs within the Figures and Supplementary Figures are provides as Supplementary Data. All molecular data is available on the NCBI database under BioProject PRJNA1163081.

Research involving human participants, their data, or biological material

Policy information about studies with human participants or human data. See also policy information about sex, gender (identity/presentation), and sexual orientation and race, ethnicity and racism.

Reporting on sex and gender	Human participants and human data are not involved in this study.
Reporting on race, ethnicity, or other socially relevant groupings	Human participants and human data are not involved in this study.
Population characteristics	Human participants and human data are not involved in this study.
Recruitment	Human participants and human data are not involved in this study.
Ethics oversight	Human participants and human data are not involved in this study.

Note that full information on the approval of the study protocol must also be provided in the manuscript.

Field-specific reporting

Please select the one below that is the best fit for your research. If you are not sure, read the appropriate sections before making your selection.

☐ Life sciences ☐ Behavioural & social sciences ☒ Ecological, evolutionary & environmental sciences

Ecological, evolutionary & environmental sciences study design

All studies must disclose on these points even when the disclosure is negative.

Study description	We generated a high-quality reference genome (n=1) for its close relative (H. cyanoguttatus), to dissect the genetic architecture of this dental polymorphism. Then, using whole genome resequencing (n=70) across the small Cuatro Ciénegas valley where H. minckleyi is endemic, we found substantial population genomic subdivision due to geography, limited evidence of introgression, and effectively no genetic structure due to pharyngeal morphotype. Then, employing quantitative trait loci mapping on 198 F2 hybrids and genome wide association (n=56), we pinpointed a single peak in an Iroquois-related (IRX) gene cluster associated with H. minckleyi's dental divergence. We compared tooth area measurements among these H. minckleyi and 33 other species of Central American Heroine cichlids.
Research sample	This work is based on a new, high-quality reference genome of Herichthys cyanoguttatus and 90 resequenced genomes that were collected between 2000 and 2010. We aimed to provide wide coverage of the geography and diversity of individuals from the sampled locations. The H. minckleyi sampled included individuals that were either molariform (M) or papilliform (P) and collected from Juan Santos (M = 10; P = 10), Tierra Blanca (M = 10; P = 10), Mojarral Este (M = 2; P = 3), Escobedo (M = 3; P = 3), and Tio Candido (M = 2; P = 3). Individuals from Los Gatos (n = 6), Mojarral Este (n = 1), and Poza Azules (n = 6).

Sampling strategy	No statistical method was used to predetermine sample size. Sample size was determined based on previously published studies using similar methodologies in this field. All sample sizes are listed in the corresdponding figure legends or on the figures.
Data collection	Adult fish were collected in field expeditions of the Hulsey lab to Mexico between 2000 and 2010. Tissues (fin clips) were dissected with scissors and scalpels and stored in pure Ethanol before DNA extraction. Darrin Hulsey and Francisco J. García de León participated in field trips. Morphological data was generated at the University of Konstanz. Genomic libraries were prepared and generated at the University of Konstanz, the Max Planck Institute of Molecular Cell Biology and Genetics in Dresden, BGI Hong Kong, Rockefeller University and PhaseGenomics.
Timing and spatial scale	Adult fish were collected in field expeditions to Mexico from 2000 to 2010.
Data exclusions	Samples of cichlids that were sequenced but not phenotyped from field collections were not included in the phenotypic analyses. This is detailed in the Supplementary tables and text.
Reproducibility	We have not conducted replication of our analyses because of the challenges in replicating them. The combination of QTL and population GWA analyses provide inferences that compliment one another.
Randomization	Within populations individuals were included in this study randomly. Groups were defined based on sampling location and pharyngeal jaw morphotype (molariform or papilliform). All subsequent analyses were performed on individuals.
Blinding	All measurements and morphometric data were collected blind to the indentity of the individual and location or origin.

Did the study involve field work? ☒ Yes ☐ No

Field work, collection and transport

Field conditions	The field collections were made in springs and freshwater ecosystems that provide a fairly stable and constant temperature and abiotic environment.
Location	Fish were caught in Mexico using hand nets, seines or by angling from Juan Santos (26° 53.86N 102° 08.81W), Tierra Blanca (26° 52.23N 102° 08.37W), Mojarral East (26° 55.48N 102° 07.28W), Escobedo (26° 52.30N 102° 05.26W), Tio Candido (26° 52.33N 102° 04.85W), Poza Azules (26° 49.73N 102° 01.68W), Los Gatos (26° 54.89N 102° 02.54W) and the Rio Salado (27° 02.06N 101° 43.30W)
Access & import/export	Collections followed protocols approved through the University of Tennessee's Institutional Animal Care and Use Committee (IACUC). We have complied with all relevant ethical regulations for animal use. The Mexican government provided permits for collection of fish used in this study (Permiso de Pesca de Fomento 230401-613-03, 2-130409-0961, DAN-01202, and DAN-02939).
Disturbance	The extraction of the sampled individuals ensured that disturbance to field sites was minimized.

Reporting for specific materials, systems and methods

We require information from authors about some types of materials, experimental systems and methods used in many studies. Here, indicate whether each material, system or method listed is relevant to your study. If you are not sure if a list item applies to your research, read the appropriate section before selecting a response.

Materials & experimental systems

n/a	Involved in the study	
☒	☐	Antibodies
☒	☐	Eukaryotic cell lines
☒	☐	Palaeontology and archaeology
☐	☒	Animals and other organisms
☒	☐	Clinical data
☒	☐	Dual use research of concern
☒	☐	Plants

Methods

n/a	Involved in the study	
☒	☐	ChIP-seq
☒	☐	Flow cytometry
☒	☐	MRI-based neuroimaging

Animals and other research organisms

Policy information about studies involving animals; ARRIVE guidelines recommended for reporting animal research, and Sex and Gender in Research

Laboratory animals	Laboratory animals were raised in the animal research facility of the University of Konstanz. S All animals were kept on a constant 12:12 light-dark cycle and at a constant temperature of 28 degrees Celsius. At no time were fish held on any kind of caloric restrictions. Sex of fishes was not associated with the phenotypes examined. The 192 fish were used to quantify the QTL of tooth areas. Laboratory strains of Herichthys cyanoguttatus were established prior to this study. At no time were fish held on any kind of

	caloric restrictions. Animal husbandry was approved by the German authorities (permit number G19-051, Regierungspräsidium Freiburg, Abteilung 3, Referat 34, Veterinärwesen & Lebensmittelüberwachung, Germany)
Wild animals	This study did not involve experimentation on wild animals.
Reporting on sex	Sex and gender considerations of the fish were not relevant to the study. The tooth phenotypes examined have been shown to be unrelated to sex.
Field-collected samples	Field Collections were made according to relevant guidelines approved by University of Konstanz in Germany.
Ethics oversight	Euthanization of animals and animal husbandry were approved by the German authorities (permit number G19-051, Regierungspräsidium Freiburg, Abteilung 3, Referat 34, Veterinärwesen & Lebensmittelüberwachung, Germany)

Note that full information on the approval of the study protocol must also be provided in the manuscript.

Plants

Seed stocks	*Report on the source of all seed stocks or other plant material used. If applicable, state the seed stock centre and catalogue number. If plant specimens were collected from the field, describe the collection location, date and sampling procedures.*
Novel plant genotypes	*Describe the methods by which all novel plant genotypes were produced. This includes those generated by transgenic approaches, gene editing, chemical/radiation-based mutagenesis and hybridization. For transgenic lines, describe the transformation method, the number of independent lines analyzed and the generation upon which experiments were performed. For gene-edited lines, describe the editor used, the endogenous sequence targeted for editing, the targeting guide RNA sequence (if applicable) and how the editor was applied.*
Authentication	*Describe any authentication procedures for each seed stock used or novel genotype generated. Describe any experiments used to assess the effect of a mutation and, where applicable, how potential secondary effects (e.g. second site T-DNA insertions, mosiacism, off-target gene editing) were examined.*

April 2023

This checklist template is licensed under a Creative Commons Attribution 4.0 International License, which permits use, sharing, adaptation, distribution and reproduction in any medium or format, as long as you give appropriate credit to the original author(s) and the source, provide a link to the Creative Commons license, and indicate if changes were made. The images or other third party material in this article are included in the article's Creative Commons license, unless indicated otherwise in a credit line to the material. If material is not included in the article's Creative Commons license and your intended use is not permitted by statutory regulation or exceeds the permitted use, you will need to obtain permission directly from the copyright holder. To view a copy of this license, visit http://creativecommons.org/licenses/by/4.0/
